# DDN3.0: determining significant rewiring of biological network structure with differential dependency networks

**DOI:** 10.1093/bioinformatics/btae376

**Published:** 2024-06-20

**Authors:** Yi Fu, Yingzhou Lu, Yizhi Wang, Bai Zhang, Zhen Zhang, Guoqiang Yu, Chunyu Liu, Robert Clarke, David M Herrington, Yue Wang

**Affiliations:** Department of Electrical and Computer Engineering, Virginia Polytechnic Institute and State University, Arlington, VA 22203, United States; Department of Electrical and Computer Engineering, Virginia Polytechnic Institute and State University, Arlington, VA 22203, United States; Department of Electrical and Computer Engineering, Virginia Polytechnic Institute and State University, Arlington, VA 22203, United States; Department of Electrical and Computer Engineering, Virginia Polytechnic Institute and State University, Arlington, VA 22203, United States; Department of Pathology, Johns Hopkins Medical Institutions, Baltimore, MD 21231, United States; Department of Automation, Tsinghua University, Beijing 100084, P.R. China; Department of Psychiatry, SUNY Upstate Medical University, Syracuse, NY 13210, United States; The Hormel Institute, University of Minnesota, Austin, MN 55912, United States; Department of Internal Medicine, Wake Forest University, Winston-Salem, NC 27157, United States; Department of Electrical and Computer Engineering, Virginia Polytechnic Institute and State University, Arlington, VA 22203, United States

## Abstract

**Motivation:**

Complex diseases are often caused and characterized by misregulation of multiple biological pathways. Differential network analysis aims to detect significant rewiring of biological network structures under different conditions and has become an important tool for understanding the molecular etiology of disease progression and therapeutic response. With few exceptions, most existing differential network analysis tools perform differential tests on separately learned network structures that are computationally expensive and prone to collapse when grouped samples are limited or less consistent.

**Results:**

We previously developed an accurate differential network analysis method—differential dependency networks (DDN), that enables joint learning of common and rewired network structures under different conditions. We now introduce the DDN3.0 tool that improves this framework with three new and highly efficient algorithms, namely, unbiased model estimation with a weighted error measure applicable to imbalance sample groups, multiple acceleration strategies to improve learning efficiency, and data-driven determination of proper hyperparameters. The comparative experimental results obtained from both realistic simulations and case studies show that DDN3.0 can help biologists more accurately identify, in a study-specific and often unknown conserved regulatory circuitry, a network of significantly rewired molecular players potentially responsible for phenotypic transitions.

**Availability and implementation:**

The Python package of DDN3.0 is freely available at https://github.com/cbil-vt/DDN3. A user’s guide and a vignette are provided at https://ddn-30.readthedocs.io/.

## 1 Introduction

**Figure 1.**
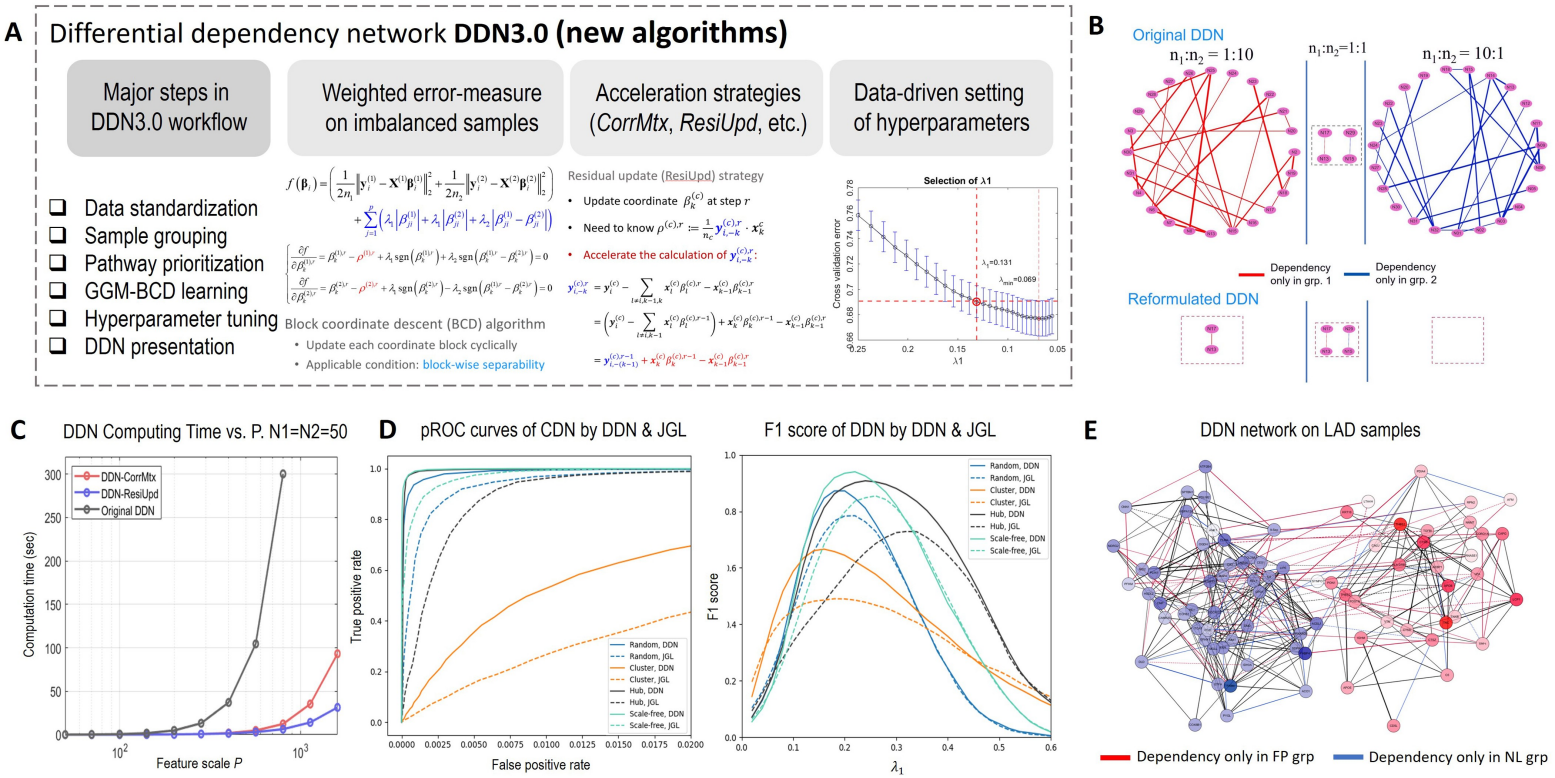
Overview of DDN3.0 tool. (A) Schematic representation of DDN3.0 workflow (overall pipeline and three new algorithms). (B) Unbiased and more accurate DDN3.0 inference (bottom portion) by the weighted error measure on balanced and imbalance samples (with size ratios of 1:10, 1:1, and 10:1), as compared with the previous biased DDN inference (top portion), under the null hypothesis. (C) Computational time saving by three newly proposed accelerating strategies. (D) Comparative evaluation of DDN3.0 and benchmark JGL measured by pROC and F1 score. (E) Interpretable biomedical case study on coronary proteomes showing significant changes in mitochondrial protein association structures between atherosclerotic and normal tissues.

Significant rewiring of regulatory association structures in pathway networks provides critical information for studying complex diseases and their progression, particularly when phenotypic transitions are involved (Mitra *et al.* 2013, Hu *et al.* 2016, Wang *et al.* 2021). Differential network analysis identifies, in a study-specific and often unknown conserved regulatory circuitry, a network of differentially wired molecular features under different conditions (Fig. 1) (Zhang *et al.* 2009, Zhang and Wang 2010). Such differential dependency networks (DDNs) are typically used to infer subtle yet relevant pathways and generate new hypotheses for further investigations (Luscombe *et al.* 2004, Zhang *et al.* 2016, Herrington *et al.* 2018). This is significant because classic differential analysis alone may miss subtle signaling in regulatory mechanisms. For example, Hudson *et al.* reported that the myostatin gene containing a causal mutation was not detected because myostatin was not differentially expressed at any of ten developmental time points under surveillance (Hudson *et al.* 2009).

Various statistical models have been proposed to infer biological network structures from data. Under a Markov random field with potentials defined on every node and edge of a network, the Gaussian Graphical Model (GGM) is widely adopted due to its many attractive properties (Meinshausen and Bühlmann 2006, Zhang *et al.* 2009). The GGM is particularly suitable for network structure inference because the problem now becomes estimating the precision matrix that concisely reflects the graphical network structure. More importantly, the edge parameters of a sparse GGM have a quantifiable relationship with conditional dependence or partial correlation among collectively predictive variables and can be efficiently estimated by Lasso regression (Zhang and Wang 2010). This is biologically plausible because GGM can mathematically represent a sparse pathway network involving causal and master regulators, in contrast to co-expression or correlation networks that may not reflect topological structure. To infer network rewiring under different conditions, two GGMs may be separately estimated and network rewiring is then detected using a permutation-based significance test (Zhang *et al.* 2009, Ottenbros *et al.* 2021). However, in addition to the computational complexity, this approach would be problematic when between-group inconsistencies are significant due to limited or imbalanced sample sizes under individual conditions and thus hinder an accurate detection of common and rewired structures (Zhang and Wang 2010).

Here, we introduce DDN3.0, an open-source software tool to infer common and rewired molecular network structures applicable to many omics data types. To accomplish this, we improved our previously developed method—DDN, which uses fused Lasso regression to jointly learn the common and rewired network structures (Zhang and Wang 2010, Tian *et al.* 2014), with three new and highly efficient algorithms (unbiased model estimation with a weighted error-measure applicable to imbalanced sample groups, multiple acceleration strategies to improve learning efficiency, and data-driven determination of proper hyperparameters) (Fig. 1A). We demonstrate the effectiveness and utility of these new functions in DDN3.0 using both realistic simulations and biomedical case studies, showing improved accuracy of differential network analysis compared with benchmark methods.

## 2 Materials and methods

### 2.1 Review of the DDN framework and previous applications

Here, we consider the problem of simultaneously learning the common structure and rewiring of a GGM between two conditions. Let a set of *p* molecular variables under two conditions be denoted by data matrices $X^{\left(1\right)}$ and $X^{\left(2\right)}$, with *N*_1_ and *N*_2_ samples, respectively. We formulate DDN inference via a fused Lasso regression and solve the following convex optimization problem for each node (molecular variable) $X_{i}^{\left(1\right)}$ and $X_{i}^{\left(2\right)}$, *i *=* *1, 2, …, *p*;

#(1) βi=argminβi=βi(1),βi(2)⁡12yi-Xβi22+λ1∑j=1pβji1+βji2+λ2βi(1)-βi(2)1,

where $\beta_{i}$ is the Lasso regression coefficient vector at node *i*, $\lambda_{1}$ and $\lambda_{2}$ are the L1 regularization hyperparameters on common and differential edges, respectively. The L2-loss function (1st term) and the L1-regularization term weighted by $\lambda_{1}$ (2nd term), lead to the reconstruction of a common and sparse graph structure. The L1-regularization term weighted by $\lambda_{2}$ (3rd term) encourages reliable detection of sparse network rewiring between two conditions, reducing false positives due to data inconsistencies (Zhang and Wang 2010). If $\beta_{ji}$ is zero, then the two variables are conditionally independent given the observations of other nodes, yet a nonzero $\beta_{ji}$ corresponds quantitatively to the partial correlation and precision matrix. Furthermore, the block-wise separability of the hybrid regularization term ensures the convergence of the block coordinate descent (BCD) learning algorithm to a globally optimum solution. We and others have previously demonstrated the performance and utility of the DDN tool on gene expression (Zhang *et al.* 2009, Tian *et al.* 2014), proteomics (Zhang *et al.* 2016, Herrington *et al.* 2018), and metabolomics data (Ottenbros *et al.* 2021).

Below we describe briefly the principles of three newly developed core algorithms in DDN3.0, namely, weighted error measure, algorithmic acceleration strategies, and data-driven determination of proper hyperparameters (Supplementary Section S2). For readers interested in the mathematical formulation, algorithmic workflow, and comparative evaluations of the DDN approach versus peer methods, we recommend the original reports (Zhang *et al.* 2009, Zhang and Wang 2010, Tian *et al.* 2014) and comprehensive reviews (Mitra *et al.* 2013, Hu *et al.* 2016, Wang *et al.* 2021).

### 2.2 Weighted error-measure on imbalance groups to infer unbiased GGMs

In our previous DDN applications to imbalanced sample groups under the null hypothesis, more falsely rewired edges were detected in the group with a smaller sample size than in the group with a larger sample size (Fig. 1B). With a closer look into the total error measure in (1), we recognized that the sparsity penalty terms are implicitly scaled by the number of samples in each group, respectively. Thus, the impact of the penalty depends on the sample size, resulting in imbalanced network structures associated with the imbalanced sample sizes.

In DDN3.0, to obtain unbiased GGM estimates where the falsely rewired edges of the common network are expected to be evenly distributed between two imbalanced groups, we propose to reformulate the original objective function by assigning a sample-size-dependent normalization factor to the error measure on each group, which effectively equalizes the contributions of different groups to the overall error-measure (Fig. 1A). Accordingly, we replace the square error term in the original Lasso with the mean squared error. Note that this normalized Lasso objective function is now independent of sample sizes.

### 2.3 Algorithmic acceleration strategies to improve learning efficiency

In our previous DDN-BCD algorithm, a significant number of inner product computations were executed. In DDN3.0, we propose to replace these inner products among data vectors with the pre-calculated equivalent and corresponding correlation coefficients, termed BCD-CorrMtx. Second, the BCD algorithm updates one coordinate at a time and thus the updated regression coefficients overlap significantly with those in the previous iteration. We propose to update the residuals in a new iteration from the previous one by merely adding two weighted data vectors, termed BCD-ResiUpd. Experimental results show that BCD-CorrMtx is suitable for large sample sizes while BCD-ResiUpd is suitable for larger networks (Fig. 1A). Third, regulatory association structures are often highly sparse, the Strong rule for pre-discarding predictors is adopted to further shrink the predictor pool in the Lasso-regression learning. Applicability of the strong rule is supported by the fact that a cross-validation strategy is used to determine the value of $\lambda_{1}$ prior to the BCD algorithm. Last, in the neighborhood selection, the solutions of the Lasso-regressions are independent among individual nodes. Thus, parallel computing is ideally applicable to the BCD algorithm and is adopted in DDN3.0.

### 2.4 Data-driven determination of proper hyperparameters

In our previous DDN framework, we used a manual selection of the two hyperparameters $\lambda_{1}$ and $\lambda_{2}$. In DDN3.0, we provide two additional options, namely, combinatorial cross-validation search and false-positive rate (FPR) control. Specifically, using 5-/10-fold cross-validation, the minimum point of the mismatch between the predicted and observed values suggests the optimal hyperparameters. Alternatively, two hyperparameters may be chosen by

#(2) λ1=2N1-Φα12p2,

#(3) λ2=12e4N−3Φ-11-α22-1e4N−3Φ-11-α22+1-11-ρ1ρ2¯,

where $\alpha_{1}$ is the FPR of the common network, $\alpha_{2}$ is the FPR of the differential network, *N* is the total number of samples, $\Phi \left(\cdot \right)$ is the cumulative distribution function of the standard normal distribution, and $\bar{\rho_{1}\rho_{2}}$ is the mediating variable of the BCD algorithm (Meinshausen and Bühlmann 2006, Zhang and Wang 2010, Tian *et al.* 2014) (Supplementary Section S2.7).

## 3 Results

### 3.1 Validation on realistic simulation data

We assess the performance of these new functions using realistic simulations. Using well-controlled simulation studies under the null hypothesis, we first assessed the effectiveness of the weighted error measures for unbiased model estimation by comparing the false rewiring distributions between the two conditions with and without the weighting, where samples were randomly divided into two groups with varying size ratios of 1:10, 1:1, and 10:1, respectively (Fig. 1B and Supplementary Section S3.1). The comparative experimental results show that the reformulated DDN3.0 objective function successfully corrects the systematic detection bias from imbalanced data and also produces much fewer false positives (Fig. 1B). We then evaluated the time saving achieved by the accelerating strategies against previous DDN using simulation data with varying numbers of samples or features (Supplementary Table S1 and Supplementary Section S3.2). The comparative experimental results show that BCD-CorrMtx is particularly efficient for larger sample sizes (Supplementary Fig. S1), BCD-ResiUpd is highly efficient for both larger sample and feature sizes (Fig. 1C), and parallel computing and the strong rule further speed up the BCD algorithm (Supplementary Tables S1 and S2, Supplementary Fig. S2). We further assessed whether the hyperparameter determination process is sensitive to changes in the dataset and/or modeling assumptions. The changes considered in the simulation studies include sample sizes and edge weights in the dataset and distribution deviation from the normal assumption. The experimental results show the robustness of the hyperparameter determination process with respect to changes in the dataset and/or modeling assumptions (Supplementary Section S5 and Supplementary Fig. S17).

### 3.2 Comparative evaluation on diverse simulation data

We compared both the accuracy and efficiency of DDN3.0 and the two most relevant benchmark methods [joint graph learning (JGL) and DINGO] on ground truth-embedded diverse simulation data (Danaher *et al.* 2014, Ha *et al.* 2015, Ottenbros *et al.* 2021). The diverse simulations cover random, cluster, hub, and scale-free (with 4, 8, and 16 modules) network types, 100 versus 400 samples, 100–400 nodes, and balanced versus imbalanced groups. We proposed four quantitative and holistic performance measures on both common and rewired structures, namely, partial receiver operating characteristic curve, precision-recall curve, F1 score, and computational time. The experimental results show that DDN3.0 consistently outperforms the JGL method with higher accuracy and efficiency by all four performance measures (Fig. 1D, Supplementary Figs S6–S13, Supplementary Tables S3–S5, Supplementary Section S4) (Danaher *et al.* 2014). DDN3.0 also outperforms the separated graph learning DINGO method again with higher accuracy and efficiency by all four performance measures (Supplementary Figs S11 and S12, Supplementary Tables S3) (Ha *et al.* 2015) (Supplementary Section S4.5).

### 3.3 Interpretable biomedical case study

We applied DDN3.0 to the human arterial proteomic features strongly associated with early atherosclerosis from 200 arterial specimens (Herrington *et al.* 2018). Differential network analysis of coronary and aortic proteomes documents significant changes in mitochondrial protein correlation structures between atherosclerotic and normal tissues, and between coronary and aortic samples, and indicates divergent mitochondrial dynamics in the setting of early atherosclerosis. Further analysis of *n* = 26 rewiring hub proteins reveals significant enrichment of tricarboxylic acid proteins (*P *=* *4.8 × 10^–6^), and major alterations in cellular necrosis factor, insulin receptor, and peroxisome proliferator-activated receptor-α/γ protein networks (Fig. 1E) (Herrington *et al.* 2018). Additional analysis reconfirms the important roles of low-density lipoproteins (LDL) and collagen proteins in disease progression from normal tissue to fibrous plaque, characterized by the enriched rewiring of the LXR/RXR activation and collagen system pathways (Supplementary Fig. S3). More details about this application and other applications of DDN3.0 can be found in Supplementary Sections S3.3–S3.5.

## 4 Discussion

In this application note, we present DDN3.0 as an efficient, accurate, and broadly applicable differential network analysis tool. Powered by the fused-Lasso regression learning framework, DDN3.0 enables joint learning of common and rewired network structures under different conditions. More importantly, the hub nodes identified by DDN3.0 provide complementary and systematic latent features for a more comprehensive enrichment analysis that can boost both the sensitivity and specificity of inferring subtle pathways, while classic differential analysis alone may overlook hidden signals in upstream regulations (Zhang *et al.* 2016, Herrington *et al.* 2018). Key features that distinguish DDN3.0 from previous work include three novel and highly efficient algorithms that make it effective to examine larger and more complex datasets yet achieve improved accuracy (Danaher *et al.* 2014, Ha *et al.* 2015, Hu *et al.* 2016, Wang *et al.* 2021).

We emphasize that biased DDN inference for imbalanced groups is a unique yet complex problem and is theoretically expected. More precisely, for GGM inference under a single condition, squared error or mean squared error measure would not produce different outcomes. However, for DDN inference between two conditions, only the mean squared error measure can theoretically ensure an unbiased inference for imbalanced groups. We are also prototyping the iDDN tool to integrate multi-omics data for modeling biological systems. Such an extension introduces new opportunities since detecting both intra- and inter-omics network structure and its rewiring can provide a more comprehensive understanding of the regulatory networks involved in disease progression. Representative preliminary results of iDDN use are reported in Supplementary Section S6.

Concerning the limitations of the current DDN3.0 tool, our future work will focus on improving both the workflow and software of iDDN and uncertainty assessment of inferred GGMs. Specifically, we will introduce plausible constraints onto the regression models to reflect not only the central dogma hierarchy but also newly discovered regulatory players and exploit a leave-one-out bootstrapping scheme to select the most reliable edge inference.

## Supplementary Material

btae376_Supplementary_Data

## Data Availability

No new data were generated or analysed in support of this research.
