# Supplementary material for: DDN3.0: determining significant rewiring of biological network structure with differential dependency networks
Source: Bioinformatics. 2024 Jun 20;40(6):btae376. doi: 10.1093/bioinformatics/btae376 (PMC11199198; doi:10.1093/bioinformatics/btae376)
Supplement: btae376_Supplementary_Data [file btae376_supplementary_data.docx]

Supplementary Information

**DDN3.0: Determining significant rewiring of** **biological network structure with** **differential dependency networks**

Yi Fu, Yingzhou Lu, Yizhi Wang, Bai Zhang, Zhen Zhang, Guoqiang Yu, Chunyu Liu, Robert Clarke, David M. Herrington, and Yue Wang

Contents

[1 Review of DDN Methods 3](#_Toc166058676)

[1.1 Review of previous DDN development 4](#_Toc166058677)

[1.2 Review of mathematical formulation in previous DDN frameworks 4](#_Toc166058678)

[2 Motivations and detailed descriptions of the novel algorithms developed in DDN3.0 6](#_Toc166058679)

[2.1 Weighted error measure for unbiased model estimation in DDN3.0 6](#_Toc166058680)

[2.2 Multiple acceleration strategies to improve learning efficiency in DDN3.0 7](#_Toc166058681)

[2.3 Accelerated BCD algorithm using correlation matrix in DDN3.0 9](#_Toc166058682)

[2.4 Accelerated BCD algorithm using residual updating strategy in DDN3.0 10](#_Toc166058683)

[2.5 Accelerated BCD algorithm by integrating the Strong Rule in DDN3.0 10](#_Toc166058684)

[2.6 Accelerated BCD algorithm with parallel computing in DDN3.0 11](#_Toc166058685)

[2.7 Guideline for hyperparameter settings in DDN3.0 11](#_Toc166058686)

[3 Results on DDN3.0 correctness, efficiency, and biomedical applications 13](#_Toc166058687)

[3.1 Weighted error-measure for unbiased model estimation in DDN3.0 13](#_Toc166058688)

[3.2 Computation time comparison on simulated data (w/ versus w/o acceleration strategies) 14](#_Toc166058689)

[3.3 DDN3.0 analysis of human arterial proteomics data from the GPAA study 16](#_Toc166058690)

[3.4 DDN3.0 analysis of ovarian cancer proteomics data from the CPTAC study 19](#_Toc166058691)

[3.5 DDN3.0 analysis of mRNA data derived from psychotic disorders 21](#_Toc166058692)

[4 Experimental comparison with peer methods 23](#_Toc166058693)

[4.1 Evaluating performance on various graph topologies and sample sizes 24](#_Toc166058694)

[4.2 Evaluating performance with different feature numbers. 26](#_Toc166058695)

[4.3 Evaluating performance on the impact of imbalance sample groups. 27](#_Toc166058696)

[4.4 Comparison of DDN3.0, DINGO, and JGL 28](#_Toc166058697)

[4.5 Comparison of DDN3.0, DINGO, and JGL on computational efficiency 31](#_Toc166058698)

[4.6 Additional comparison of DDN3.0 and DDN2.0 on computational efficiency 33](#_Toc166058699)

[5 Sensitivity of hyperparameter determination 35](#_Toc166058700)

[6 Prototype of the application on multi-omics data 37](#_Toc166058701)

[7 Discussion 39](#_Toc166058702)

[8 Python Package 40](#_Toc166058703)

[References 41](#_Toc166058704)

# Review of DDN Methods

Complex diseases are often characterized by misregulation of relevant biological pathways (Bandyopadhyay, et al., 2010; Barabasi, et al., 2011). Causal rewiring of regulatory structures in multi-omics networks provides critical information for studying complex diseases and their progression, particularly when phenotypic transitions are involved (Califano, 2011; Hu, et al., 2016; Mitra, et al., 2013). We used the differential dependency network (DDN) tool to identify, from within a complex and often unknown regulatory circuitry, a network of rewired molecular nodes between grouped samples (Tian, et al., 2015; Wang, et al., 2021). DDN discovers the differentially-networked latent features that can boost both the sensitivity and specificity of detecting major causal pathways because classic differential analysis alone may overlook subtle changes in upstream regulations (Creixell, et al., 2012; Herrington, et al., 2018; Reverter, et al., 2010; Zhang, et al., 2016).

DDN infers network structures by estimating conditional dependencies among genes. Conditional dependency is a key type of probabilistic relationship that is distinct from the basic correlation relationship. If two genes with correlated expressions are conditionally independent, their expression levels will be uncorrelated after accounting for all other genes’ expressions. Conditional dependence relationship hence is less likely to reflect transitional effects than basic correlation relationship, and provides stronger evidence of functional relationships between genes. These functional relationships could be regulatory or other molecular interactions that cause the two genes’ expressions to be tightly coupled.

DDN uses local dependency models to characterize the dependencies among genes in the network and represent local network structures. Unlike pairwise correlation, the conditional dependence between two genes cannot be measured solely based on the expression levels of these two genes. Rather, all possible links to all other genes should be considered to find the network that best explains the expression data. DDN adapts an efficient neighborhood selection strategy based on a Lasso regression to enable such inference. The permutation test is introduced in DDN to evaluate empirical p-values of the detected network rewiring (Tian, et al., 2011). The detected differential edges with multi-test corrected p-values less than the preset significance threshold (e.g., p-value<0.05) are marked as significant network rewiring.

## Review of previous DDN development

Here we review the underlying conceptual framework for DDN and summarize our previous work to develop DDN as a tool for differential network analysis. Various approaches for differential network analysis may be divided into two major categories (Hu, et al., 2016; Mitra, et al., 2013; Wang, et al., 2021; Zhang, et al., 2014): (1) two GGMs are separately estimated using condition-specific samples, followed by a differential analysis of edges to detect network rewiring (Class, et al., 2018; Ha, et al., 2015; He, et al., 2019; Ottenbros, et al., 2021; Zhang, et al., 2009); (2) the common and differential edges of two GGMs are jointly estimated using all samples while adopting a fused-Lasso formulation (Danaher, et al., 2014; Tian, et al., 2014; Zhang and Wang, 2010; Zhao, et al., 2014).

We first introduced DDN1.0 that belonged to category #1, where each GGM was estimated using a Lasso-regression learning strategy (Zhang, et al., 2009; Zhang, et al., 2011). We and others have recognized that while learning the graphical models under two conditions can be separately achieved and the structural and parametric differences can be subsequently compared, this technically convenient framework can collapse when the structural and parametric inconsistencies from limited data samples and noise effects are significant and hinder an accurate detection of true and meaningful structural and parametric changes.

We then introduced DDN2.0 that belonged to category #2, by formulating the joint GGM learning as a fused Lasso-regression problem (Zhang and Wang, 2010). We discussed the properties of the problem formulation and introduced an efficient block coordinate descent (BCD) algorithm. We demonstrated the principle of the approach on a numerical simulation experiment, and we then applied the algorithm to the modeling of gene regulatory networks under different conditions and obtained promising yet biologically plausible results. We further integrated prior knowledge into DDN2.0 framework (Tian, et al., 2014; Tian, et al., 2011), and this knowledge-fused DDN2.0 method is implemented as an open-source Cytoscape app called kDDN (Tian, et al., 2015).

## Review of mathematical formulation in previous DDN frameworks

For DDN1.0, the objective function is, for each node, finding the Lasso regression coefficients of the following optimization problem (Zhang, et al., 2009; Zhang, et al., 2011):

$$\left\{ \begin{aligned} \boldsymbol{\beta}_{i}^{\left( 1 \right)}=\mathrm{argmin}_{\boldsymbol{\beta}_{i}^{\left( 1 \right)}} f\left( \boldsymbol{\beta}_{i}^{\left( 1 \right)} \right)=\frac{1}{2}\left\| \boldsymbol{y}^{\left( 1 \right)}-\boldsymbol{X}^{\left( 1 \right)}\boldsymbol{\beta}_{i}^{\left( 1 \right)} \right\|_{2}^{2}+\lambda\left| \boldsymbol{\beta}_{i}^{\left( 1 \right)} \right| \\ \boldsymbol{\beta}_{i}^{\left( 2 \right)}=\mathrm{argmin}_{\boldsymbol{\beta}_{i}^{\left( 2 \right)}} f\left( \boldsymbol{\beta}_{i}^{\left( 2 \right)} \right)=\frac{1}{2}\left\| \boldsymbol{y}^{\left( 2 \right)}-\boldsymbol{X}^{\left( 2 \right)}\boldsymbol{\beta}_{i}^{\left( 2 \right)} \right\|_{2}^{2}+\lambda\left| \boldsymbol{\beta}_{i}^{\left( 2 \right)} \right| \end{aligned} \right.,$$

where $\boldsymbol{X}^{\left( 1 \right)}$ and $\boldsymbol{X}^{\left( 2 \right)}$ are the observed data matrix under condition 1 and condition 2; $\boldsymbol{y}^{\left( 1 \right)}$ and $\boldsymbol{y}^{\left( 2 \right)}$ are the observed data vector of the *i-*th node under two conditions; $\boldsymbol{\beta}_{i}^{\left( 1 \right)}$ and $\boldsymbol{\beta}_{i}^{\left( 2 \right)}$ are the Lasso regression coefficients, also denoted as local neighborhood structure in which the non-zero elements represent the structural connection between the corresponding node and the *i-*th node. After the local neighborhood structures $\boldsymbol{\beta}_{i}$ are learned for all nodes, they are merged to form the adjacency or precision matrix which represents the network structure.

For DDN2.0, we assume that the network structures between the two conditions share a large portion of common network structures, and the network rewiring events are sparse. Thus, we improved DDN1.0 by jointly solving Lasso regressions while introducing a penalty term on the structural difference, namely fused Lasso regressions (Zhang and Wang, 2010). The DDN2.0 optimization problem becomes:

$$\boldsymbol{\beta}_{i}=\left( \boldsymbol{\beta}_{i}^{\left( 1 \right)},\boldsymbol{\beta}_{i}^{\left( 2 \right)} \right)=\mathrm{argmin}_{\boldsymbol{\beta}_{i}} f\left( \boldsymbol{\beta}_{i} \right)=\frac{1}{2}\left\| \boldsymbol{y}_{i}^{\left( 1 \right)}-\boldsymbol{X}^{\left( 1 \right)}\boldsymbol{\beta}_{i} \right\|_{2}^{2}+\lambda_{1}\sum_{j=1}^{p} \left( \left| \beta_{ji}^{\left( 1 \right)} \right|+\left| \beta_{ji}^{\left( 2 \right)} \right| \right)+\lambda_{2}\left\| \boldsymbol{\beta}_{i}^{\left( 1 \right)}-\boldsymbol{\beta}_{i}^{\left( 2 \right)} \right\|_{1}$$

where *i* is the node index; *β_ji_* is the regression coefficient from node *j* to node *i*; ***y****_i_* and ***X*** are the expression values of dependent and input variables, respectively; *P* is the number of nodes other than $i$; *λ*_1_ and *λ*_2_ are the hyperparameters on the two penalty terms that are used to assure both a sparse common network structure and sparse differential network rewiring.

For kDDN, we proposed to integrate prior knowledge into DDN2.0 (Tian, et al., 2015). It has a better graphical user interface and is very convenient for data visualization. Specifically, the kDDN optimization problem is:

$$\boldsymbol{\beta}_{i}=\left( \boldsymbol{\beta}_{i}^{\left( 1 \right)},\boldsymbol{\beta}_{i}^{\left( 2 \right)} \right)=\mathrm{argmin}_{\boldsymbol{\beta}_{i}} f\left( \boldsymbol{\beta}_{i} \right)=\frac{1}{2}\left\| \boldsymbol{y}_{i}-\boldsymbol{X}\boldsymbol{\beta}_{i} \right\|_{2}^{2}+\lambda_{1}\sum_{j=1}^{P} \left( 1-W_{ji}\theta\right)\left( \left| \beta_{ji}^{\left( 1 \right)} \right|+\left| \beta_{ji}^{\left( 2 \right)} \right| \right)+\lambda_{2}\left\| \beta_{ji}^{\left( 1 \right)}-\beta_{ji}^{\left( 2 \right)} \right\|_{1}$$

where *W* is the matrix of fused prior knowledge of node-to-node connections, such as protein-protein interactions in pathways; and θ is a weighting parameter (Tian, et al., 2014).

# Motivations and detailed descriptions of the novel algorithms developed in DDN3.0

Below we describe in detail the principles and pipelines of three newly developed core algorithms in DDN3.0: (i) unbiased model estimation with a weighted error measure applicable to imbalance sample groups, (ii) multiple acceleration strategies to improve learning efficiency, and (iii) data-driven determination of proper hyperparameters.

## Weighted error measure for unbiased model estimation in DDN3.0

From the application of DDN2.0 with various data sets, we noticed a potential systematic bias - in imbalanced data, which refers to the dataset with unequal numbers of samples in each group, DDN2.0 consistently detects more differential edges in the condition with a smaller sample size than the other condition with a larger sample size, as illustrated in Fig. 1B using well-controlled simulation data. By taking a closer look into the objective function of DDN2.0 (Zhang and Wang, 2010), we recognized its dependency on the sample size *N* and/or the feature size *P*. The penalty is the sum of absolute values of all regression coefficients, and hence is independent of the sample size. Conversely, the error measure is positively correlated with the sample size for the standardized predictor variables. However, the objective function does not explicitly assign a weight to each group, which gives equal weights to both case and control groups (Tian, et al., 2014). We emphasize that biased DDN inference for imbalanced groups is a unique yet complex problem and theoretically is expected. More precisely, for GGM inference under a single condition, squared error or mean squared error measure would not make any difference. However, for DDN inference between two conditions, only the mean squared error measure can theoretically ensure an unbiased inference for imbalanced groups.

We rewrite the objective function as:

$$f\left( \boldsymbol{\beta}_{i} \right)=\frac{1}{2}\left\| \boldsymbol{y}_{\boldsymbol{i}}-\boldsymbol{X}\boldsymbol{\beta}_{i} \right\|_{2}^{2}+\lambda_{1}\left( \left| \boldsymbol{\beta}_{i}^{\left( 1 \right)} \right|+\left| \boldsymbol{\beta}_{i}^{\left( 2 \right)} \right| \right)+\lambda_{2}\left\| \boldsymbol{\beta}_{i}^{\left( 1 \right)}-\boldsymbol{\beta}_{i}^{\left( 2 \right)} \right\|_{1}$$

$$=\frac{1}{2}\left[ \sum_{l=1}^{n_{1}} \left( x_{i,l}-\sum_{k=1}^{P} x_{k,l}\beta_{ik}^{\left( 1 \right)} \right)^{2}+\sum_{l=n_{1}+1}^{n_{1}+n_{2}} \left( x_{i,l}-\sum_{k=1}^{P} x_{x,l}\beta_{ik}^{\left( 2 \right)} \right)^{2} \right]+\left[ \lambda_{1}\left| \boldsymbol{\beta}_{i}^{\left( 1 \right)} \right|+\lambda_{1}\left| \boldsymbol{\beta}_{i}^{\left( 2 \right)} \right| \right]+\lambda_{2}\left\| \boldsymbol{\beta}_{i}^{\left( 1 \right)}-\boldsymbol{\beta}_{i}^{\left( 2 \right)} \right\|_{1}$$

$$=\left[ \frac{1}{2}\sum_{l=1}^{n_{1}} \left( x_{i,l}-\sum_{k=1}^{P} x_{k,l}\beta_{ik}^{\left( 1 \right)} \right)^{2}+\lambda_{1}\left| \boldsymbol{\beta}_{i}^{\left( 1 \right)} \right| \right]+\left[ \frac{1}{2}\sum_{l=n_{1}+1}^{n_{1}+n_{2}} \left( x_{i,l}-\sum_{k=1}^{P} x_{x,l}\beta_{ik}^{\left( 2 \right)} \right)^{2}+\lambda_{1}\left| \boldsymbol{\beta}_{i}^{\left( 2 \right)} \right| \right]+\lambda_{2}\left\| \boldsymbol{\beta}_{i}^{\left( 1 \right)}-\boldsymbol{\beta}_{i}^{\left( 2 \right)} \right\|_{1}.$$

$$=\sum_{l=1}^{n_{1}} \left[ \frac{1}{2}\left( x_{i,l}-\sum_{k=1}^{P} x_{k,l}\beta_{ik}^{\left( 1 \right)} \right)^{2}+\frac{\lambda_{1}}{n_{1}}\left| \boldsymbol{\beta}_{i}^{\left( 1 \right)} \right| \right]+\sum_{l=n_{1}+1}^{n_{1}+n_{2}} \left[ \frac{1}{2}\left( x_{i,l}-\sum_{k=1}^{P} x_{x,l}\beta_{ik}^{\left( 2 \right)} \right)^{2}+\frac{\lambda_{1}}{n_{2}}\left| \boldsymbol{\beta}_{i}^{\left( 2 \right)} \right| \right]+\lambda_{2}\left\| \boldsymbol{\beta}_{i}^{\left( 1 \right)}-\boldsymbol{\beta}_{i}^{\left( 2 \right)} \right\|_{1}$$

We can see that, while the weights for each group are equal, *i.e.,* the sample-wise squared residual is at a comparable level, the actual weighted penalty of beta added to each sample differs. When minimizing the total object function with the L1 penalty, samples in the group with smaller sizes are subject to penalties different from the samples in the group with more samples, which leads to imbalanced network structures between the two groups.

Accordingly, we proposed the objective function of DDN3.0 using the normalized Lasso objective function, and hence make it independent of the sample sizes:

$$\tilde{f}\left( \boldsymbol{\beta} \right)=\tilde{f}_{1}\left( \boldsymbol{\beta}_{i}^{\left( 1 \right)} \right)+\tilde{f}_{2}\left( \boldsymbol{\beta}_{i}^{\left( 2 \right)} \right)+\lambda_{2}\left\| \boldsymbol{\beta}_{i}^{\left( 1 \right)}-\boldsymbol{\beta}_{i}^{\left( 2 \right)} \right\|_{1}$$

$$=\frac{1}{2}\left[ \frac{1}{n_{1}}\sum_{l=1}^{n_{1}} \left( x_{i,l}-\sum_{k=1}^{P} x_{k,l}\beta_{ik}^{\left( 1 \right)} \right)^{2}+\frac{1}{n_{2}}\sum_{l=n_{1}+1}^{n_{1}+n_{2}} \left( x_{i,l}-\sum_{k=1}^{P} x_{x,l}\beta_{ik}^{\left( 2 \right)} \right)^{2} \right]+\lambda_{1}\sum_{j=1}^{P} \left( \left| \beta_{ji}^{\left( 1 \right)} \right|+\left| \beta_{ji}^{\left( 2 \right)} \right| \right)+\lambda_{2}\left\| \boldsymbol{\beta}_{i}^{\left( 1 \right)}-\boldsymbol{\beta}_{i}^{\left( 2 \right)} \right\|_{1}.$$

The above formulation is expected to be unbiased or less biased.

## Multiple acceleration strategies to improve learning efficiency in DDN3.0

The block coordinate descent (BCD) approach was proposed by Banerjee, et al. (2008) for solving the Lasso problem efficiently. Friedman, et al. (2008) showed with experiments that a coordinate descent procedure for the graphical Lasso problem is 30-4000 times faster than competing methods, making it computationally attractive. Note that the objective function of DDN3.0 has block-wise separability and can be expressed as the sum of two parts. The first part is convex and differentiable. While the second part is convex and non-differentiable, it is the summation of several non-overlapping members. We rewrite the objective function as follows:

$$f\left( \boldsymbol{\beta}_{i} \right)=\left( \frac{1}{2n_{1}}\left\| y_{i}^{\left( 1 \right)}-X^{\left( 1 \right)}\boldsymbol{\beta}_{i}^{\left( 1 \right)} \right\|_{2}^{2}+\frac{1}{2n_{2}}\left\| y_{i}^{\left( 2 \right)}-X^{\left( 2 \right)}\boldsymbol{\beta}_{i}^{\left( 2 \right)} \right\|_{2}^{2} \right)+\sum_{j=1}^{p} \left( \lambda_{1}\left| \beta_{ji}^{\left( 1 \right)} \right|+\lambda_{1}\left| \beta_{ji}^{\left( 2 \right)} \right|+\lambda_{2}\left| \beta_{ji}^{\left( 1 \right)}-\beta_{ji}^{\left( 2 \right)} \right| \right)$$

The second part of the objective function can be written as the sum of *p* terms with non-overlapping members $\left( \beta_{j}^{\left( 1 \right)},\beta_{j}^{\left( 2 \right)} \right), j=1,\cdots,p$. Each $\left( \beta_{j}^{\left( 1 \right)},\beta_{j}^{\left( 2 \right)} \right), j=1,\cdots,p$, is called a coordinate block. Tseng (2001) proved that, if one function is a block-wise separable convex function, its global minimum could be reached by repeated application of block-wise optimization with the cyclic rule, and convergence is guaranteed.

The essence of the BCD algorithm is a “one-block-at-a-time” scheme (Tian, et al., 2011; Zhang and Wang, 2010). At iteration $r+1$, only one coordinate block, $\left( \beta_{k}^{\left( 1 \right)},\beta_{k}^{\left( 2 \right)} \right)$, is updated, with the remaining $\left( \beta_{j}^{\left( 1 \right)},\beta_{j}^{\left( 2 \right)} \right),j\in\left\{ 1,\cdots k-1,k+1,\cdots,p \right\}$ fixed at their values. The cyclic rule is used to update parameter estimation iteratively, *i.e.,* update the parameter pair $\left( \beta_{j}^{\left( 1 \right)},\beta_{j}^{\left( 2 \right)} \right)$ for each $j=1,\cdots,p$, one by one in a circular way in the iterations. The figure below illustrates the solution subregions of $\beta_{k}^{\left( 1 \right),r}$and $\beta_{k}^{\left( 2 \right),r}$ on the plane of $\left( \rho^{\left( 1 \right),r},\rho^{\left( 2 \right),r} \right)$. For readers interested in the mathematical details and algorithmic workflows of the DDN framework, we highly recommend the original reports (Tian, et al., 2014; Zhang, et al., 2009; Zhang and Wang, 2010).


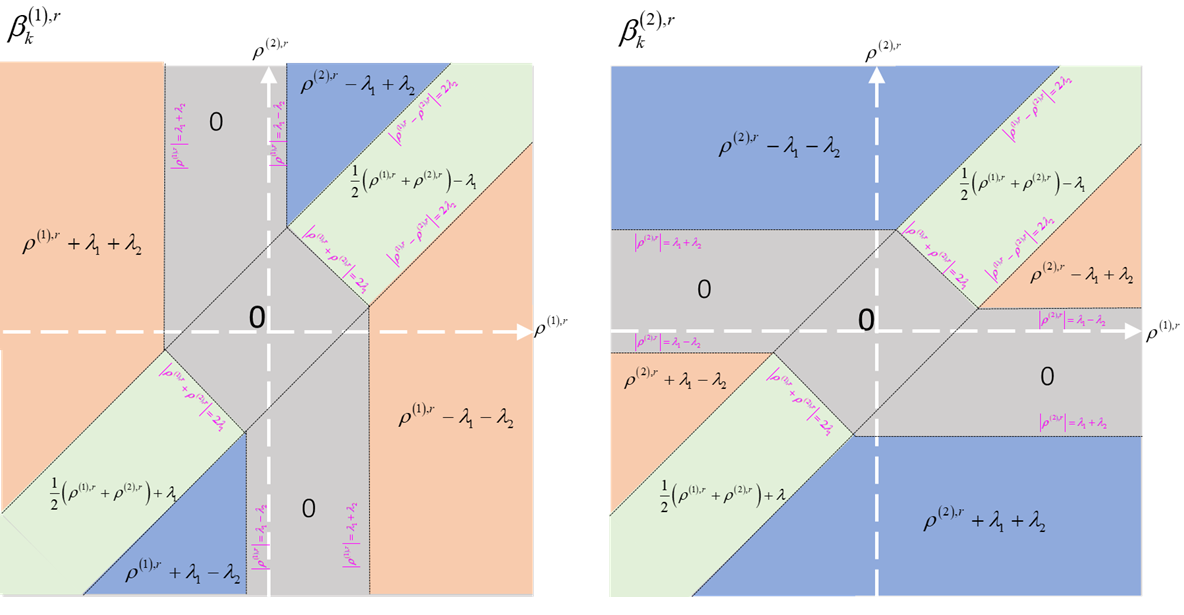


## Accelerated BCD algorithm using correlation matrix in DDN3.0

The BCD algorithm uses the cyclic rule to update the coefficients for each coordinate block in a cyclic round, and repeat the cyclic round iteratively until convergence. Assuming convergence is reached after *T* times of cyclic rounds on average, the DDN method in total needs $TP^{2}$ times of coefficient updating. In the original form of $\rho^{\left( 1 \right),r}$ and $\rho^{\left( 2 \right),r}$, calculating the residuals $y_{i,-k}^{r}$ takes about *PN* times of multiplication, the inner product of the residual vector and the current response variable vector requires additional *N* times of multiplication. Thus, each BCD update needs approximately $2\times\left( PN+N \right)\cong2PN$ times of multiplication, leading to the computational complexity of DDN-BCD learning to be $O\left( TP^{2}\times2PN \right)=O\left( 2P^{3}N \right)$.

The calculation of $\rho^{\left( 1 \right),r}$ and $\rho^{\left( 2 \right),r}$ takes the form of an inner product between the residual vector and the current node’s observed data vector,

$$\rho^{\left( 1 \right),r}=\frac{1}{n_{1}}y_{i,-k}^{\left( 1 \right),r}*x_{k}^{\left( 1 \right)}$$

$$=\frac{1}{n_{1}}\left( x_{i}^{\left( 1 \right)}*x_{k}^{\left( 1 \right)}-\sum_{l\neq i,k} \beta_{l}^{\left( 1 \right),r}x_{l}^{\left( 1 \right)}*x_{k}^{\left( 1 \right)} \right)$$

$$=R_{ik}^{\left( 1 \right)}-\sum_{l\neq i,k} \beta_{l}^{\left( 1 \right),r}R_{lk}^{\left( 1 \right)}$$

$$=R_{ik}^{\left( 1 \right)}-\sum_{l\neq i,k} \beta_{l}^{\left( 1 \right),r-1}R_{lk}^{\left( 1 \right)}$$

$$=-\tilde{\beta}^{\left( 1 \right),r-1}*R_{\cdot k}^{\left( 1 \right)}$$

$$\rho^{\left( 2 \right),r}=-\tilde{\beta}^{\left( 2 \right),r-1}*R_{\cdot k}^{\left( 2 \right)}.$$

Therefore, we can replace the inner product of the observed data by the pre-calculated correlation coefficients. Specifically, $\rho^{\left( 1 \right),r}$and $\rho^{\left( 2 \right),r}$ is directly updated from $\beta^{r-1}$ and the pre-calculated correlation matrix $R^{\left( 1 \right)}$ and $R^{\left( 2 \right)}$. This strategy is termed the DDN-BCD-CorrMtx, reducing the computational complexity to $O\left( TP^{2}\times2P \right)=O\left( 2TP^{3} \right)$ and achieving an approximately *N* times speed-up.

## Accelerated BCD algorithm using residual updating strategy in DDN3.0

Recalling that the BCD algorithm updates one coordinate at a time, thus the updated $\beta^{r}$ will have most of its elements overlapped with those of $\beta^{r-1}$ in the previous iteration as follows,

$$y_{i,-\left( k+1 \right)}^{\left( 1 \right),r+1}= \text{y}_{i}^{\left( 1 \right)}-\sum_{l\neq i,k+1} x_{l}^{\left( 1 \right)}\beta_{l}^{\left( 1 \right),r+1}$$

$$=y_{i}^{\left( 1 \right)}-\sum_{l\neq i,k,k+1} x_{l}^{\left( 1 \right)}\beta_{l}^{\left( 1 \right),r+1}-x_{k}^{\left( 1 \right)}\beta_{k}^{\left( 1 \right),r+1}$$

$$=y_{i}^{\left( 1 \right)}-\sum_{l\neq i,k} x_{l}^{\left( 1 \right)}\beta_{l}^{\left( 1 \right),r}+x_{k+1}^{\left( 1 \right)}\beta_{k+1}^{\left( 1 \right),r}-x_{k}^{\left( 1 \right)}\beta_{k}^{\left( 1 \right),r}$$

$$=y_{i,-k}^{\left( 1 \right),r}+x_{k+1}^{\left( 1 \right)}\beta_{k+1}^{\left( 1 \right),r}-x_{k}^{\left( 1 \right)}\beta_{k}^{\left( 1 \right),r}\text{, for }r\geq1.$$

Instead of re-calculating the residuals from *P* weighted observed vectors, we can update the new residuals based on the previous values with a small computation load by adding two weighted observation vectors. This strategy is termed the DDN-BCD-ResiUpd, reducing the computational complexity to $O\left( TP^{2}\times5N \right)=O\left( 5TNP^{2} \right)$, approximately 0.4*P* times faster.

## Accelerated BCD algorithm by integrating the Strong Rule in DDN3.0

The Strong rules are based on the “Safe” rules proposed by El Ghaoui et al. (2010) and are able to effectively reduce the actual number of predictors that need to be solved in Lasso problems. Tibshirani, et al. (2012) showed that, although in extremely rare cases the Strong rules may erroneously discard predictors, the error could be amended by checking the KKT conditions. The basic Strong rule is defined as follows: for the lasso problem, discard the $j$-th predictor from the optimization problem if:

$$\left| x_{j}^{T}y \right|<2\lambda-\lambda_{\text{max}},$$

where $\lambda_{\text{max}}={max}_{j} \left| x_{j}^{T}y \right|$ is the smallest tuning parameter value such that $\hat{\beta}\left( \lambda_{\text{max}} \right)=0$. For the fused Lasso regression in DDN3.0, we use a cross-validation strategy to determine $\lambda_{1}$ while setting $\lambda_{2}=0$. Accordingly, we apply the Strong rule to pre-discard predictors before executing the BCD algorithm. The figure below illustrates the effectiveness of the Strong rule (variable pre-screening) on a toy dataset for computational savings.


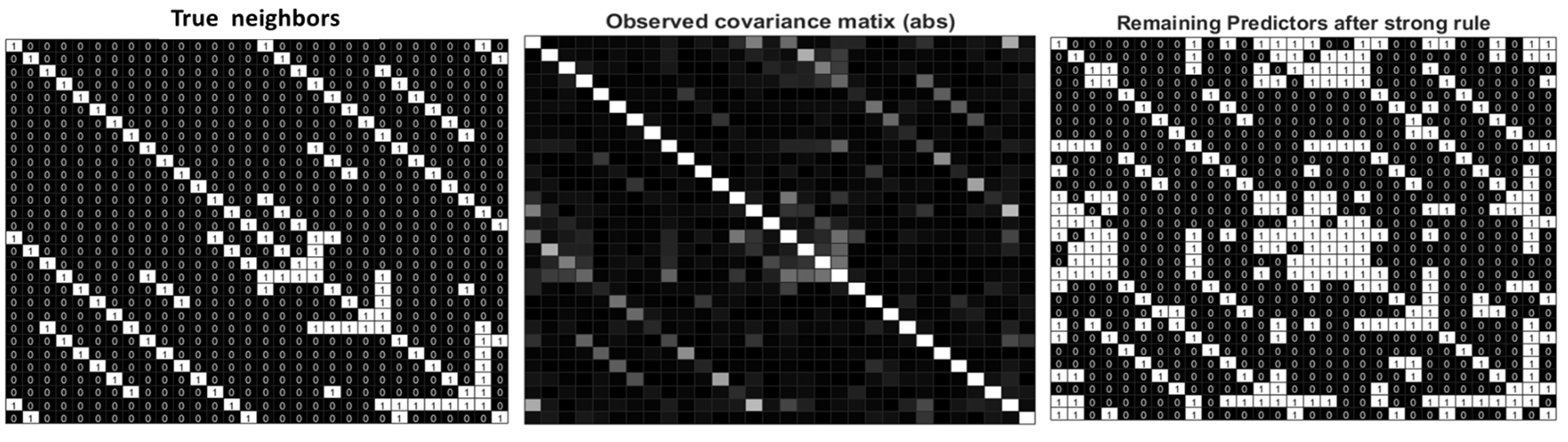


## Accelerated BCD algorithm with parallel computing in DDN3.0

In the neighborhood selection approach, the solution of one node’s optimization is independent of the other nodes’ solution. Therefore, the BCD optimization could work in parallel for the $P$ nodes in the network. In parallel computing, we assign one CPU core from a multi-core computer to independently solve one node’s DDN3.0 optimization problem. The full DDN3.0 network construction could be about *N_core* times fast, where *N_core* is the number of available CPU cores.

We developed a Python package to implement parallel computing along with other accelerating methods of DDN-CorrMtx or DDN-ResiUpd. We denote the methods with parallel computing as DDN-CorrMtx-Parallel and DDN-ResiUpd-Parallel. In the test on simulated data, we found that parallel computing significantly increases the speed of the BCD algorithm, particularly in the case of a large feature size *P*.

## Guideline for hyperparameter settings in DDN3.0

There are three hyperparameter tuning options for DDN3.0 users, namely, manual setting, cross-validation, and false-positive rate (FPR) control. Firstly, besides the manual setting, we use a cross-validation strategy to choose $\lambda_{1}$ and then determine the value of $\lambda_{2}$. If we temporarily set $\lambda_{2}$ as zero, the differential network analysis is simplified to standard GGM network inference, and the neighborhood selection for each node is equivalent to a standard Lasso regression, and hence we may use the same cross-validation strategy as discussed in (Friedman, et al., 2017) to choose $\lambda_{1}$ that minimizes the cross-validation error. One standard error rule (Friedman, et al., 2017) could optionally be used as the rule of thumb to increase robustness. In addition, for smaller networks and sample sizes, the two parameters could be determined by grid search and joint crossvalidation.

Secondly, we determine the value of$\lambda_{2}$ for a given significance level. From the solution subregions in the plane of $\left( \rho_{1},\rho_{2} \right)$ we can see that $\beta_{k}^{\left( 1 \right),r}$and $\beta_{k}^{\left( 2 \right),r}$will be identical in the subregion of $\left| \rho_{1}-\rho_{2} \right|<2\lambda_{2}$. Therefore, the question becomes what value of $\left| \rho_{1}-\rho_{2} \right|<2\lambda_{2}$ is considered significantly large enough, at a given significance level? Since $X_{1}$ and $X_{2}$ follow a multivariate Gaussian distribution, we could apply Fisher’s transform to correlation coefficients $\rho_{1}$and $\rho_{2}$ for standardized. Define:

$$z_{1}=\frac{1}{2}\ln\frac{1+\rho_{1}}{1-\rho_{1}},z_{2}=\frac{1}{2}\ln\frac{1+\rho_{2}}{1-\rho_{2}}.$$

$z_{1}$ and $z_{2}$, as the results of Fisher’s transform, closely approximates the Gaussian distributions of $G\left( \frac{1}{2}\ln\frac{1+\bar{\rho}_{1}}{1-\bar{\rho}_{1}},\frac{1}{n_{1}-3} \right)$ and $G\left( \frac{1}{2}\ln\frac{1+\bar{\rho}_{2}}{1-\bar{\rho}_{2}},\frac{1}{n_{2}-3} \right)$. Since $\rho_{1}$ and $\rho_{2}$ are equal in the null hypothesis of the absence of the differential edges, $z_{1}$ and $z_{2}$ share the same mean. If independence is assumed between $\rho_{1}$ and $\rho_{2}$, we could infer that the variable $z=z_{1}-z_{2}$ follows a Gaussian distribution with zero-mean and its variance equal to the sum of two Gaussian random variable’s variances: $1/\left( n_{1}-3 \right)+1/\left( n_{2}-3 \right)$. For a given significance level $\alpha$ (for example, 0.05), define the significance threshold for $\left| z \right|=\left| z_{1}-z_{2} \right|$ as:

$$s\left( \alpha\right)=\sqrt{\frac{1}{n_{1}-3}+\frac{1}{n_{2}-3}}\Phi^{-1}\left( 1-\frac{\alpha}{2} \right)<\left| z \right|=\left| z_{1}-z_{2} \right|.$$

Since group imbalance was not considered in derivation of the original DDN, its significance threshold is calculated here based on a special case of $n_{1}=n_{2}=N$. In that case, $s\left( \alpha\right)=\frac{2}{\sqrt{N-3}}\Phi^{-1}\left( 1-\frac{\alpha}{2} \right)$. Rewrite the significance condition in the form of $\rho_{1}$ and $\rho_{2}$, we have:

$$\left| z \right|=\left| z_{1}-z_{2} \right|>s\left( \alpha\right)\Leftrightarrow\left| \rho_{1}-\rho_{2} \right|>\frac{e^{2s\left( \alpha\right)}-1}{e^{2s\left( \alpha\right)}+1}\left( 1-\rho_{1}\rho_{2} \right)=2\lambda_{2}.$$

Since$\lambda_{2}$ is applied for all nodes’ optimization, we replace $\rho_{1}\rho_{2}$ with the sample mean values estimated from all the samples: $\rho_{1}\rho_{2}\leftarrow\overline{\rho_{1}\rho_{2}}=2\sum_{1\leq i<j\leq p} R_{ij}^{\left( 1 \right)}R_{ij}^{\left( 2 \right)}/p\left( p-1 \right)$. Finally, we get the value of $\lambda_{2}$ under the significance level $\alpha$:

$\lambda_{2}=\frac{e^{2s\left( \alpha\right)}-1}{2\left( e^{2s\left( \alpha\right)}+1 \right)}\left( 1-\overline{\rho_{1}\rho_{2}} \right)$.

# Results on DDN3.0 correctness, efficiency, and biomedical applications

## Weighted error-measure for unbiased model estimation in DDN3.0

We designed a simulation experiment to confirm the bias brought by imbalanced data in the original DDN method and to show that our proposed reformulated DDN objective function is capable of correctly handling imbalanced data. To illustrate the systematic bias, we need to identify which differential edges are the false positive detections caused by bias. We purposely designed data that contains no structure rewiring, and hence all detected differential edges will be false positives. The simulated data is actually single condition data that follows an i.i.d. multivariate Gaussian distribution and is manually and randomly divided into two groups to mimic two pseudo conditions. We generated data with total sample size *N*=500 and feature scale *P*=30, and then divided into two groups with three settings of sample ratios: 1:1, 1:10, and 10:1. The first sample ratio of 1:1 represents the perfect balance of sample size, while the remaining two sample ratios correspond to imbalanced data. The generated data were standardized to zero-mean and unit variance, and then tested by the original DDN method and the reformulated DDN3.0.

The experimental results are shown in Fig. 1B. The condition-specific differential edges are colored for conditions 1 or 2. The edge width is mapped from p-values evaluated by a permutation test; wider edges have smaller p-values. For the data group with a balanced sample size ratio of 1:1, the original DDN and the sample-scale-normalized DDN3.0 gave identical results: only two network rewiring events with insignificant p-values were detected. These results are as expected because there should be no network rewiring in the simulated data. For imbalanced data groups with sample size ratios of 1:10 and 10:1, the original DDN gives network rewiring detection results of overwhelmingly one-sided condition-specific network rewiring. Some of the detected differential edges have significant p-values from the permutation test. Since there is no network rewiring in the designed ground truth network, all condition-specific network rewiring detected by the original DDN method are false positives, confirming the presence of systematic bias when using imbalanced data. Conversely, DDN3.0 detected only one or zero network rewiring events in these imbalanced data. The detected differential edges have insignificant p-values and could be filtered further. The results show the low false positive rate of DDN3.0 in the simulation data. Hence, the systematic bias in the original DDN method, *i.e.,* condition-specific false positives with imbalanced data, has been successfully corrected.

## Computation time comparison on simulated data (w/ versus w/o acceleration strategies)

We use a series of simulation studies to compare the computation time of DDN with the four proposed accelerating strategies in DDN3.0: DDN-CorrMtx, DDN-ResiUpd, DDN-StrongRule, and DDN-CorrMtx/ResiUpd-Parallel. To compare the methods fairly, we set the simulation conditions as follows: each simulated data has $P$ nodes and follows an i.i.d. standard Gaussian distribution; the true covariance matrix is the identity matrix, hence no edges exist in the graph and only the first round of iterations is needed for each node; $\lambda_{1}$ and$\lambda_{2}$ are also set large, therefore convergence is expected after the first round of iterations. For each case of simulated data, all methods use the same data as inputs. The testing environment is listed as follows: Intel® Core™ i5-8300H CPU @2.30Ghz; 16GB RAM; R 3.6.1. The computation time is recorded by the R function of *system.time()*.

**Table S1**. The computation time of accelerated DDN methods.

|  | N=100, P=1600 | N=1600, P=100 |
| --- | --- | --- |
| Original DDN | 2695.48s | 141.25s |
| DDN-CorrMtx | 93.19s | 0.28s |
| DDN-CorrMtx-  Parallel (nCore=7) | 1.48s | 0.25s |
| DDN-ResiUpd | 31.39s | 2.94s |

Table S1 listed two simulated cases: one with a high feature scale $P$ and the other with a high sample size $N$. From the comparison, the original DDN method takes the longest time, which is untenable in large scale. DDN3.0 with the correlation matrix strategy takes little time for a large sample size but is slower than DDN3.0 with residual updating strategy when the feature scale $P$ is large. Parallel computing offers significant benefits when $P$ is large but the time saving is limited with moderate values of $P$.

Fig. 1C in the main text shows the computation time used by DDN-CorrMtx, DDN-ResiUpd, and the original DDN, all versus the feature scale $P$. The DDN-ResiUpd method shows a large advantage when $P$ grows. Fig. S1 shows the computation time used by the three methods versus sample size $N$. Since the dimension of the correlation matrix only relies on $P$, the DDN-CorrMtx method uses almost the same time for different sample scales, and hence is much faster than the other two methods when $N$ is large.


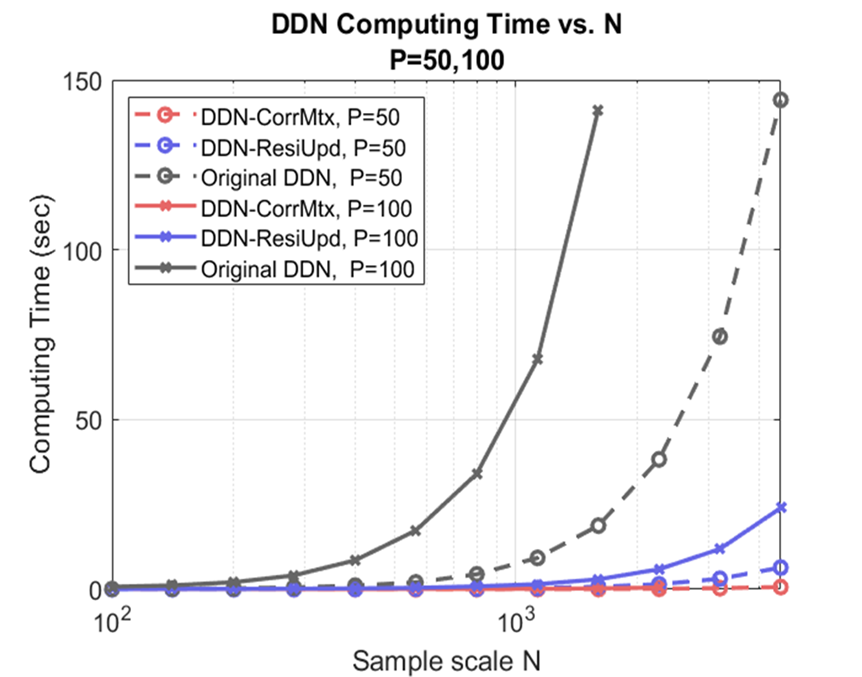


**Figure S1** The impact of sample size $N$ on the running time of DDN and its accelerated versions.

We compare the computation time of the DDN-CorrMtx methods with and without parallel computing (Fig. S2). Since parallel computing is done for each node selected from all $P$ nodes, it uses much less computation time when the feature scale $P$ is large. However, the time saved by parallel computing is limited when $P$ is small.


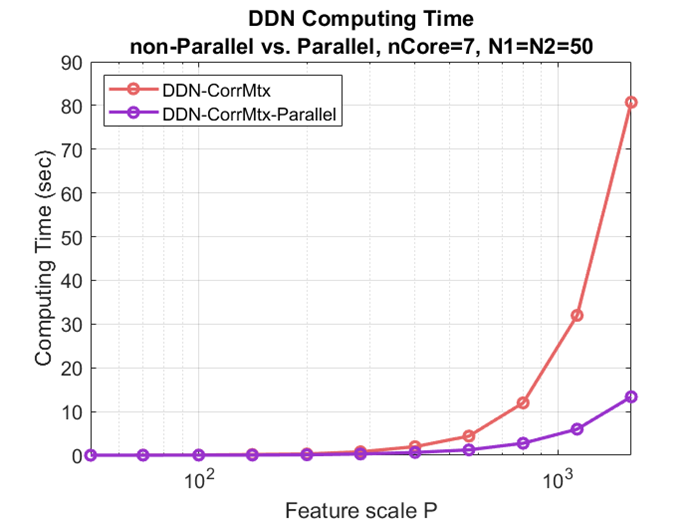


**Figure S2**. DDN computation time with and without parallel computing.

Table S2 shows the effectiveness of the Strong rule. In this simulation study, the ground truth network is designed as a single ring in which each node has exactly two neighbors. Therefore, the basic Strong rules could effectively discard most of the predictors. The results show that computation time could be significantly reduced by applying the Strong rule when the network has high sparsity.

**Table S2**. Computation time comparison between DDN with and without Strong rule.


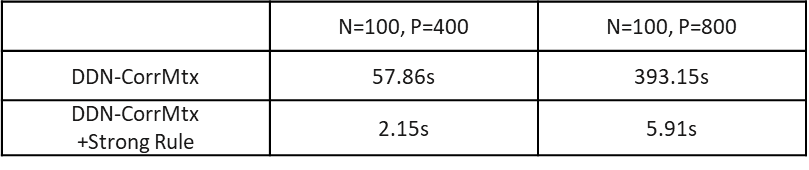


In summary, we have presented three accelerated computing methods for DDN3.0 by reformulating the BCD algorithm in DDN. We also proposed acceleration strategies of discarding predictors by the Strong rule and by parallel computing. Depending on the relative values of the sample size $N$ and the feature scale $P$, we may choose correlation matrix strategy or residual updating strategy. The results show a tremendous reduction in computation time compared with the original DDN method. The proposed DDN-ResiUpd method with parallel computing is now able to handle thousands of genes in a reasonable time period (<10 minutes), compared with the original DDN method which is only capable of handling dozens of genes.

## DDN3.0 analysis of human arterial proteomics data from the GPAA study

Atherosclerosis is defined at the molecular level as an assembly of intra- and extra-cellular proteins, which jointly alter the cellular processes and produce characteristic remodeling of the local vascular environment. In the GPAA research projects, the human arterial proteomics data are collected from the coronary artery and aortic specimens from 200 arterial specimens (Herrington, et al., 2018). The differential networks detected define the composition of the protein networks and also the regulatory features likely associated with early atherosclerosis.

The samples were collected from two anatomic locations of the left anterior artery (LAD) and distal abdominal aorta (AA). Each sample was then examined by pathologists to evaluate the proportions of four tissue types: complicated lesion (CO), fibrous plaque (FP), fatty streak (FS), and normal tissue (NL). According to the pathologist’s evaluation, no sample contains CO tissue, 66 samples were evaluated as completely normal, and the remaining samples contained certain proportions of FP and/or FS tissues. The samples can be clustered into 3 major groups by the first two principal components, namely FP, FS, and NL. Comparison between the FP group and the NL group is of most interest, since the existence of FP tissue indicates a more severe atherosclerosis. Given the fact that most samples are evaluated as completely normal, to reduce the impact of the imbalance of group sizes we further selected 30 samples from the normal group. We used the top principal component values to provide an acceptable group size ratio (2:1) with the FP group while retaining as many samples as possible.

We performed DDN3.0 analysis on LAD protein expression data of 89 differentially expressed genes identified between the FP and NL groups, and of the top significantly enriched pathways and gene groups regulated by the top upstream regulators (TNF-α, INSR, PPAR-α, PPAR-γ). Similarly for the AA samples, 80 genes were identified by GO term enrichment and IPA analysis, although the enrichment analysis did not statistically implicate inhibition of the insulin receptor, PPAR-α, or PPAR-γ pathways. DDN3.0 results show distinct network patterns and rewiring differences for the two anatomic locations (LAD and AA). Each of the activated and inhibited regulatory pathways identified in the LAD samples (TNF-α, INSR, PPAR-α, and PPAR-γ) exhibited significant re-wiring among pathway proteins between FP and NL samples. The re-wiring models reveal selective coupling or uncoupling between specific protein pairs in these regulatory networks depending on the specific tissue phenotype (FP or NL). These differential networks suggest major disruptions in energy metabolism, extracellular matrix remodeling, and PKA signal transduction when compared with normal tissue. Significant network rewiring events in the setting of fibrous plaque were detected in five additional pathways over-represented in both the LAD and AA samples (TGFB1, TP53, MEGEA5, MYC, ERBB2), see Fig. 1E (highlights: multiple significant network rewiring events; hub genes in DDN on LAD samples are found significantly enriched in TCA pathway, p=4.8E–6.).

With detected significant rewiring events both in FP-associated proteins and in candidate gene sets from IPA-picked pathways and master regulators, DDN3.0 analysis gives a comprehensive understanding of arterial protein networks and how they change in early atherosclerosis. It also suggests that the human arterial proteome can be viewed as a complex network with architectural features that vary considerably as a function of anatomic location and the presence or absence of atherosclerosis. These divergent proteomic features could have important implications for personalized treatment and prevention.


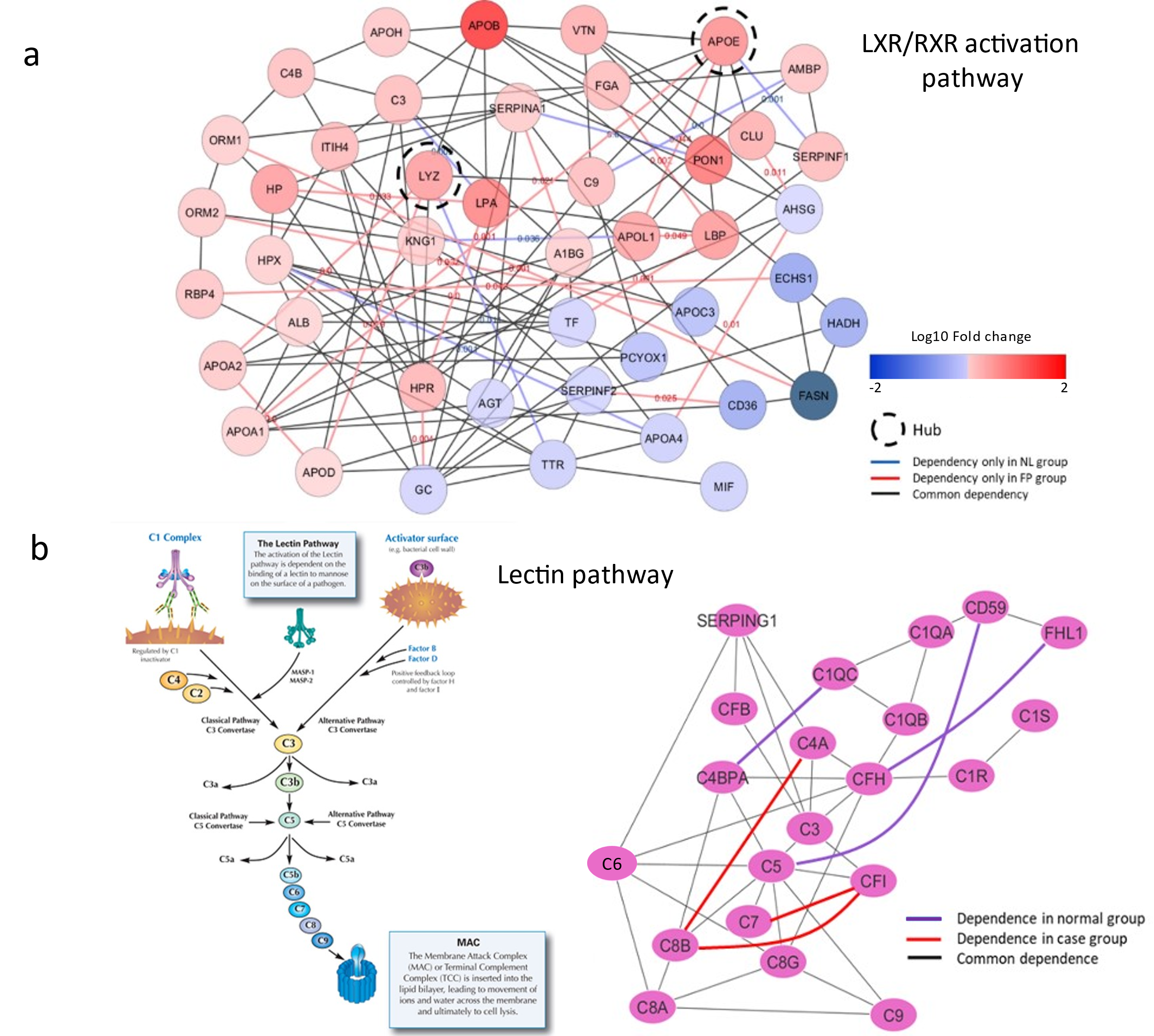


**Figure S3**. DDN results for selected genes on proteomics data from AA samples. (a) Application of DDN on genes of the LXR/RXR activation pathway. Each node represents a gene and its color coding represents the fold change from normal samples (NL group) to case samples (FP group). The weights of rewired edges are annotated on the edges. We highlight two hub genes with dashed circles. (b) Application of DDN on the Lectin pathway. The left panel gives an overview of the Lectin pathway, which is a type of cascade reaction in the complement system. The right panel gives the results of DDN, where common and differential edges are represented in different colors.

For the 89 identified FP-associated proteins, DDN detected a group of proteins pivotal in the rewiring of the network structure between NL and FP in the LAD samples. Further analysis of these 26 rewiring hub proteins revealed significant enrichment of tricarboxylic acid proteins (p-value=4.8E–6). Subsequent DIA-MS analysis of n=114 mitochondrial proteins was performed comparing FP versus NL samples from the LAD. The results document an average 60% reduction of a wide range of mitochondrial proteins in the FP samples in comparison with NL samples after adjustment for vascular smooth muscle-specific housekeeping proteins, age, and sex. This consistent reduction indicates divergent mitochondrial dynamics in the setting of atherosclerosis characterized by reduced mitochondrial mass in the LAD. In contrast, a similar analysis of the same proteins in the distal Abdominal Aorta (AA) samples revealed a much less consistent and statistically non-significant pattern of mitochondrial protein suppression. Collectively, these data demonstrate how DDN-detected network rewiring could produce biologically coherent insights that may not be evident from conventional statistical or pathway enrichment analysis strategies. We also found some protein groups play important roles in network changes from normal to disease. For example, low-density lipoproteins (LDL) and high-density lipoproteins (HDL) in the LXR/RXR activation pathway, and proteins in the Lectin pathway in the complementary system are linked with some detected network rewiring (Fig. S3).

## DDN3.0 analysis of ovarian cancer proteomics data from the CPTAC study

DDN3.0 tool is used in the CPTAC (Clinical Proteomic Tumor Analysis Consortium) project of integrated proteogenomic characterization of human high grade serous ovarian cancer (Zhang, et al., 2016). CPTAC ovarian cancer group performed a comprehensive mass-spectrometry-based proteomic characterization of 174 ovarian tumors previously analyzed by The Cancer Genome Atlas (Zhang, et al., 2016). DDN3.0 analysis was applied to 171 BRCA1/BRCA2-related proteins on global proteomics data between homologous recombination deficiency (HRD) positive and HRD negative groups. In the second stage of CPTAC, more than 100 additional samples were included in the prospective sample set. Among these new samples, 84 were high grade serous tumors and characterized with the new TMT10 labeling proteomics technique. The global protein expression data of these prospective samples once again validated the statistically significant lower acetylation levels in both K12 and K16 peptides of H4 histone.


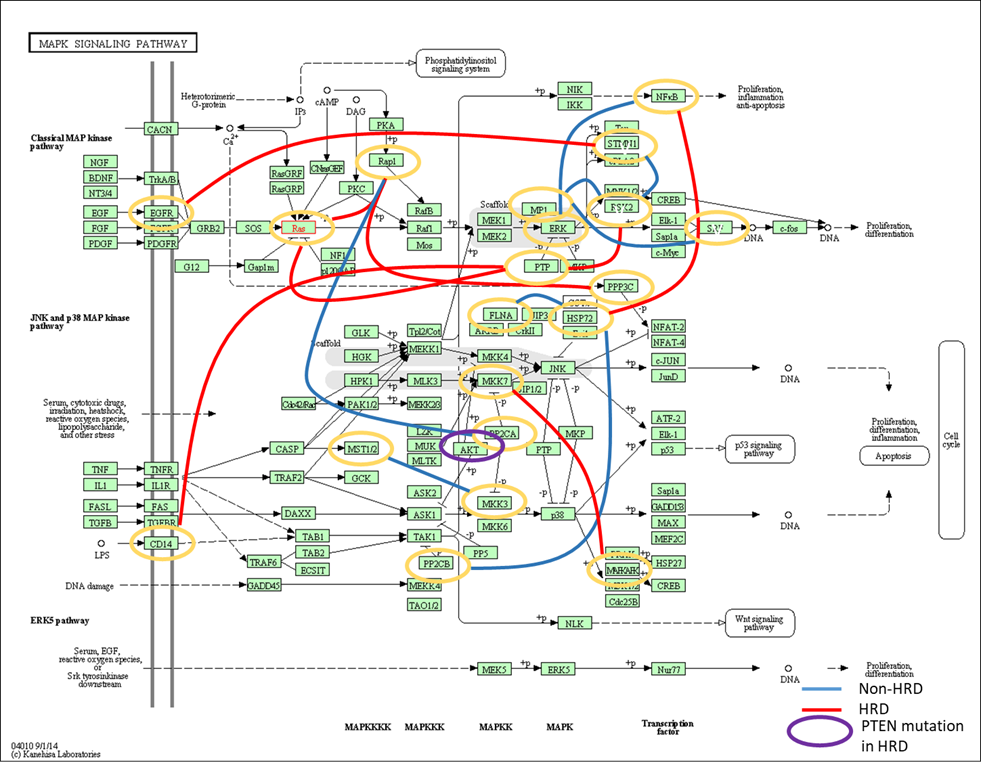


**Figure S4**. DDN network overlapped on the KEGG diagram of the MAPK signaling pathway. A subnetwork of DDN results containing proteins involved in histone acetylation is highlighted by yellow circles.

For the CPTAC retrospective sample set, homologous recombination deficiency (HRD) is defined by the presence of germline or somatic BRCA1 or BRCA2 mutations, BRCA1 promoter methylation, or homozygous deletion of PTEN. For the CPTAC prospective sample, HRD samples are classified by germline or somatic BRCA1/BRCA2/PTEN mutations. The BRCA1/BRCA2-related target genes are collected and integrated from the literature.

To select the candidate genes from the whole collection of data, we chose core pathways that have been well studied and proved to be highly related to cancer. DDN3.0 results on the MAPK signaling pathway highlighted the AKT gene as one of the hub genes pivotal in the network rewiring events. AKT is modulated by PTEN mutation that is one of the key markers of HRD status. Fig. S4 shows this network constructed on the protein expression data from 122 samples of the CPTAC retrospective dataset, with 66 HRD positive vs. 56 HRD negative sample. The results show the acetylation levels of two key peptides from histone protein H4 have a significant difference between HRD and none-HRD samples, validated by independent tests.

## DDN3.0 analysis of mRNA data derived from psychotic disorders

Schizophrenia (Schiz.) and bipolar disorder (BP) are psychiatric disorders with relatively high heritability. In addition, transcriptomes are reported to show dynamic dysregulation in the diseased brain sections (Colantuoni, et al., 2011; Kang, et al., 2011; Kuhn, et al., 2011). The Center of Depression and Resilience at the University of Illinois at Chicago conducted a research project investigating the dysregulation changes in bipolar disorder (BP) and schizophrenia (SCH) in the Cerebellum (CB) and parietal cortex (PC) brain tissues (Chen, et al., 2014). The biological samples were obtained from Neuropathology Consortium and Array collections, and the genome-wide gene expression data were tested by Affymetrix microarray chips. Samples from cerebellum (CB) and parietal cortex (PC) brain tissues were collected from 150 subjects, including 50 bipolar disorder (BP) samples, 50 schizophrenia (Schiz.) samples, and 50 unaffected (control) samples (Chen, et al., 2013). Expression data were obtained from the NCBI Gene Expression Omnibus (GEO) accession code of GSE35978. One of the two PC data sets came from SMRI samples (PFC-SMRI), and the second came from the Victorian Brain Bank Network (PFC-VBBN), with GEO code of GSE21138. Candidate gene sets for DDN analysis are selected from the top pathways picked by the IPA tool and also from the genes in two co-expression modules from WGCNA analysis.

DDN3.0 analysis showed multiple significant network rewiring events in the cell adhesion pathway for each comparison scenario (Fig. S5). These rewiring events highlight the fact that the two psychiatric disorders of interest likely involve numerous proteins and dynamic alterations in gene regulation networks rather than only one or a few isolated proteins. We also observed some highly similar patterns between Schiz. and BP in selected pathways. For example, though the gene ITGA8 is significantly differentially expressed between CB and PC samples, it shows a common network rewiring feature. Specifically, ITGA8 has disconnections for BP and Schiz. diseases in CB samples and for Schiz. in PC samples, all with significant p-values. DDN analysis also revealed some in-depth differences of network rewiring events of the two psychiatric disorders, which could be hidden in traditional differential analysis of gene expression levels.


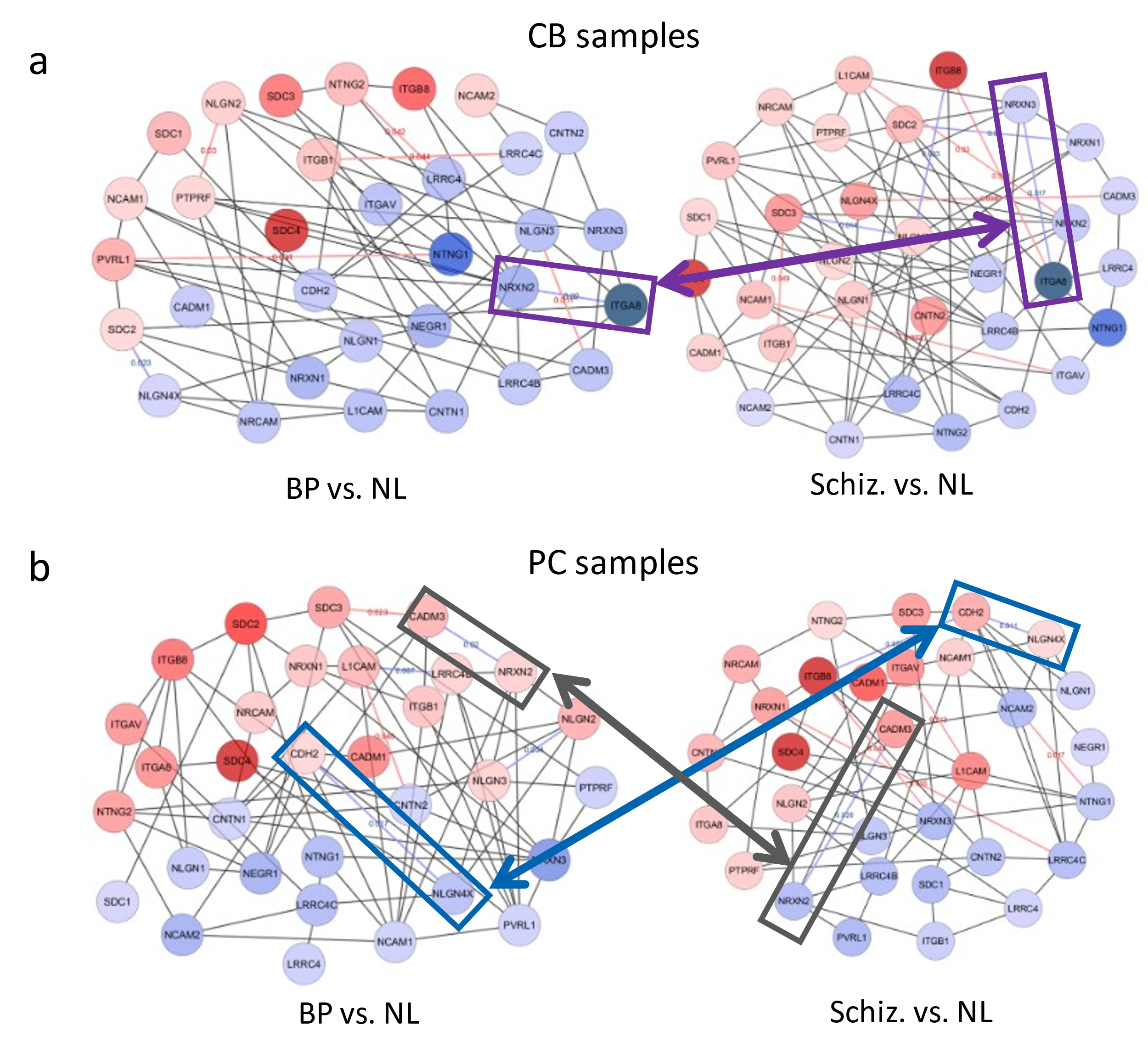


**Figure S5**. DDN3.0 detected rewiring events in the gene expression dependency network for the two psychiatric disorders (Schiz. and BP). (a) Results on the CB samples. The network on the left shows the dependency network of genes using bipolar disorder (BP) and control (NL) samples. The black edges are common for both conditions. The blue edges are the rewired edges occurring in the NL group, while the red edges are rewired in the disease group. The color of each node represents the fold change of expression of that gene in two conditions. The blue color means the expression is lower under the disease condition. Similarly, the network on the right is between Schizophrenia (Schiz.) And NL samples. The two boxes in these two networks highlight an edge that is rewired for both diseases. (b) The results on the PC samples. We highlight two edges that are rewired in these two diseases.

# Experimental comparison with peer methods

We conducted experimental comparisons between DDN3.0 and the most relevant peer methods of differential network analysis tools. We compared both the accuracy and efficiency of DDN3.0 and the two most relevant benchmark methods (JGL and DINGO) on ground truth-embedded diverse simulation data (Danaher, et al., 2014; Ha, et al., 2015; Ottenbros, et al., 2021). The diverse simulations cover random, cluster, hub, and scale-free (with 4, 8, 16 modules) network types, 100 versus 400 samples, 100 to 400 nodes, and balance versus imbalance groups. We proposed four quantitative and holistic performance measures on both common and rewired structures, namely, partial receiver operating characteristic (pROC) curve, precision-recall curve, $F_{1}$ score, and computational time.

We first discuss simulation studies that evaluates the performance of DDN 3.0 and compare it with two benchmark methods. Our focus is on the accuracy of detecting common and differential networks between two conditions. JGL jointly infers the precision matrices under two conditions by formulating the task as a maximum likelihood estimation (MLE) problem, which is solved by the ADMM algorithm. In contrast, DINGO infers the network structures in two stages: the first stage learns the common network, while the second learns the differential rewiring. Because these two methods use different principles, we compared DDN3.0 with each.

We generated the simulations data using the HUGE R package (Zhao, et al., 2012). We chose different graph topologies provided by HUGE packages and generated common networks first. For common networks, the off-diagonal value of the precision matrix was initialized as 0.5. The HUGE package changed these values to ~0.3 to ensure the precision matrix is positive definite. We generated two conditions based on this common network by adding 25% extra non-zero (and non-diagonal) elements to the precision matrix. To maintain positive definiteness, we set the weights of those extra edges to 0.9 times the mean non-diagonal values of the common precision matrix. If the procedure fails, *i.e.,* the smaller eigenvalue becomes less than zero, we sampled another set of non-zero elements.

Throughout the simulations, we use precision and recall, and TP and FP as the evaluation measures for common and differential networks. Precision was defined as the ratio of true positive edges and total detections. Recall was defined as the ratio of true positive edges with the number of all ground truth edges. TP is the number true edges detected. FP is the number of detected edges that are false. This choice is also used in the peer methods DINGO and JGL. Another widely used measure is the ROC curve, which is a reasonable choice for showing the performance of the common network but is not suitable for the differential network. Moreover, since each combination of $\lambda_{1}$ and $\lambda_{2}$ leads to several accuracy measures (precision and recall, and TP and FP, for common/differential network), it is desirable to use an overall performance measure. Here we calculate the $F_{1}$ score of the common network, and the $F_{1}$ score for the differential network. We take the average of these two scores as an overall measure. An $F_{1}$ score was defined as the harmonic mean of the precision and recall.

## Evaluating performance on various graph topologies and sample sizes

We performed simulations on various graph topologies with 100 nodes. For random graphs, we generated the Erdos-Renyi graph with 100 nodes. For cluster graphs, we simulated 5 clusters, each with 20 nodes. For hub graphs, we simulated graphs with 5 hubs, each with 20 nodes. We then simulated graphs containing four scale-free graphs, with 24 common edges in each subgraph. We also simulated graphs with one or eight scale-free subgraphs (results not shown here). For each graph, we simulated two different sample sizes. We first simulated the scenario where each of the two conditions has 200 samples; there are 400 samples in total. These scenarios mimic the cases of relatively larger sample sizes. We then simulated the cases where each condition has 50 samples, so there are 100 samples in all. This scenario simulates cases with relatively smaller sample sizes.

We evaluated the performance of methods with a large combination of the values of $\lambda_{1}$ and $\lambda_{2}$. For $\lambda_{1}$, we use values ranging from 0.02 to 1, with a step size of 0.02. Then for each $\lambda_{1}$, we evaluated seven $\lambda_{2}$ values, ranging from 0.025 to 0.15, with a step size of 0.025. Therefore, we evaluated 350 combinations. This wide choice of hyper-parameters allows us to understand the best achievable accuracies of the compared methods and to avoid the impact of non-ideal model selection methods. We repeated the simulation multiple times to make the evaluation at each combination of $\lambda_{1}$ and $\lambda_{2}$ more accurate. Specifically, if each condition has 200 samples, the simulation was repeated 20 times. If each condition has only 50 samples, we repeated 40 times. We note that in our simulation, for each repeat, we re-generate the graph from scratch to avoid the bias introduced by specific graph topology. In all these graph types, and in terms of both common and differential networks, DDN3.0 outperforms JGL in different evaluation measures, which shows DDN can achieve better performance in diverse graph types compared with peers (Fig. S6 and Fig. S7).


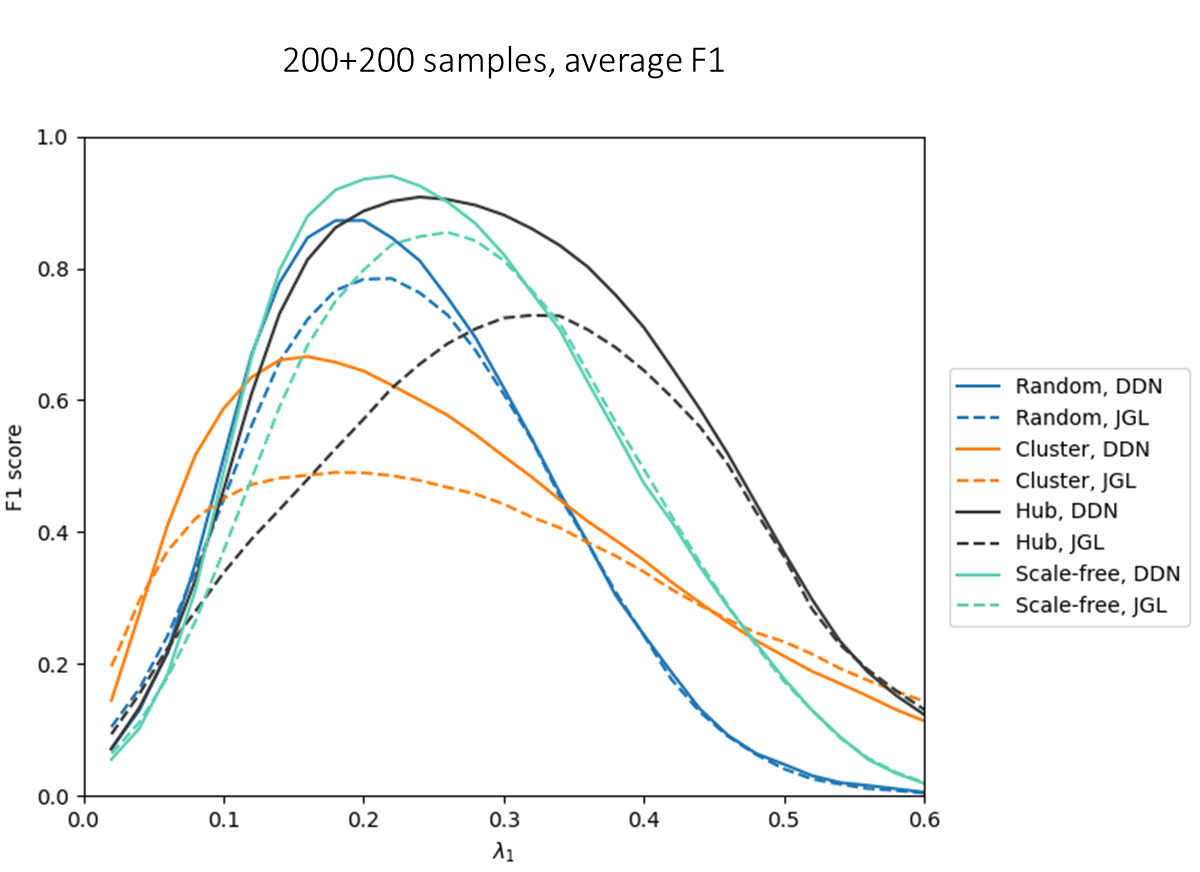


**Figure S6**. Comparative evaluation of DDN3.0 and benchmark method JGL measured by average $F_{1}$ score (sample size 200+200, same for Fig. 1D). DDN3.0 is ‘DDN’ in the figures.


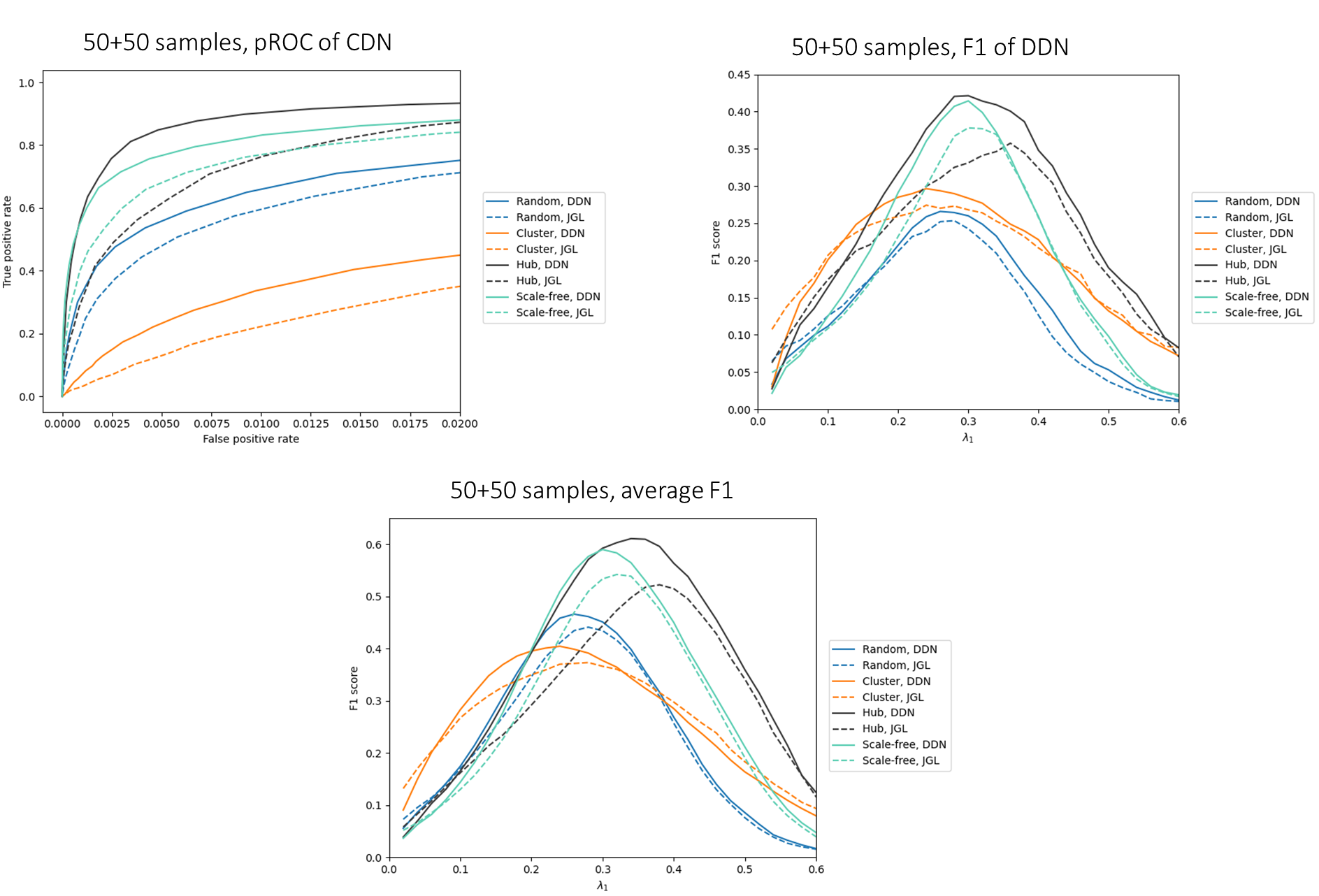


**Figure S7**. Comparative evaluation of DDN3.0 and benchmark JGL measured by pROC curve of the common network, $F_{1}$ of the rewired network, and average $F_{1}$ (sample size 50+50).

## Evaluating performance with different feature numbers.

Next, we studied the impact of feature number on performance by using graphs with different numbers of nodes. We set the node numbers to 100, 200, and 400. For 100, 200, and 400 nodes, we generated a scale-free graph with 4, 8, and 16 modules, respectively. We also show the results with two different sample sizes: 400 samples and 100 samples. DDN still outperforms JGL in all these simulation settings, showing that DDN is suitable for networks with larger feature numbers (Fig. S8 and Fig. S9).


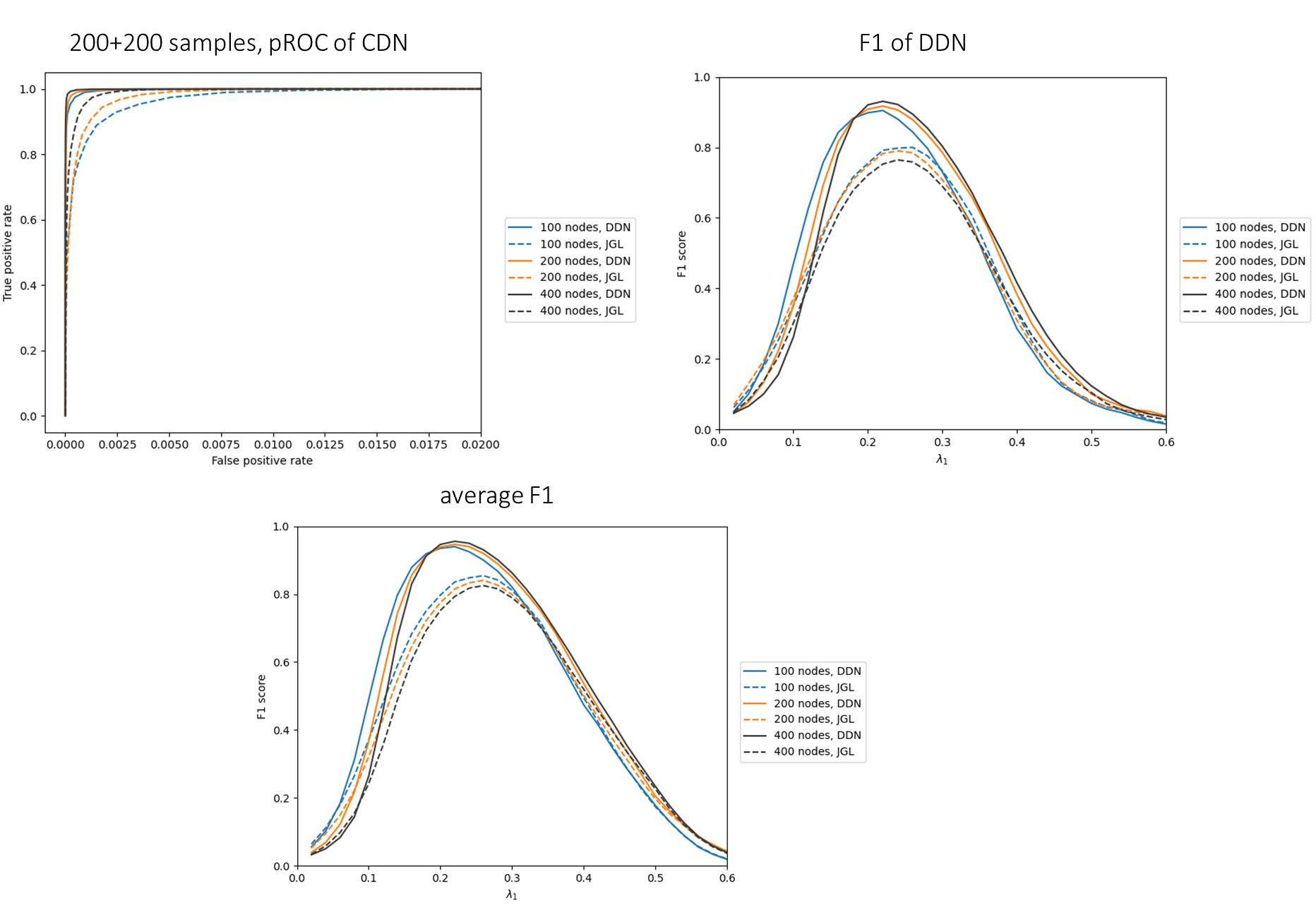


**Figure S8**. Comparative evaluation of DDN3.0 and benchmark JGL measured by pROC curve of the common network, $F_{1}$ of the rewired network, and average $F_{1}$ (sample size 200+200).


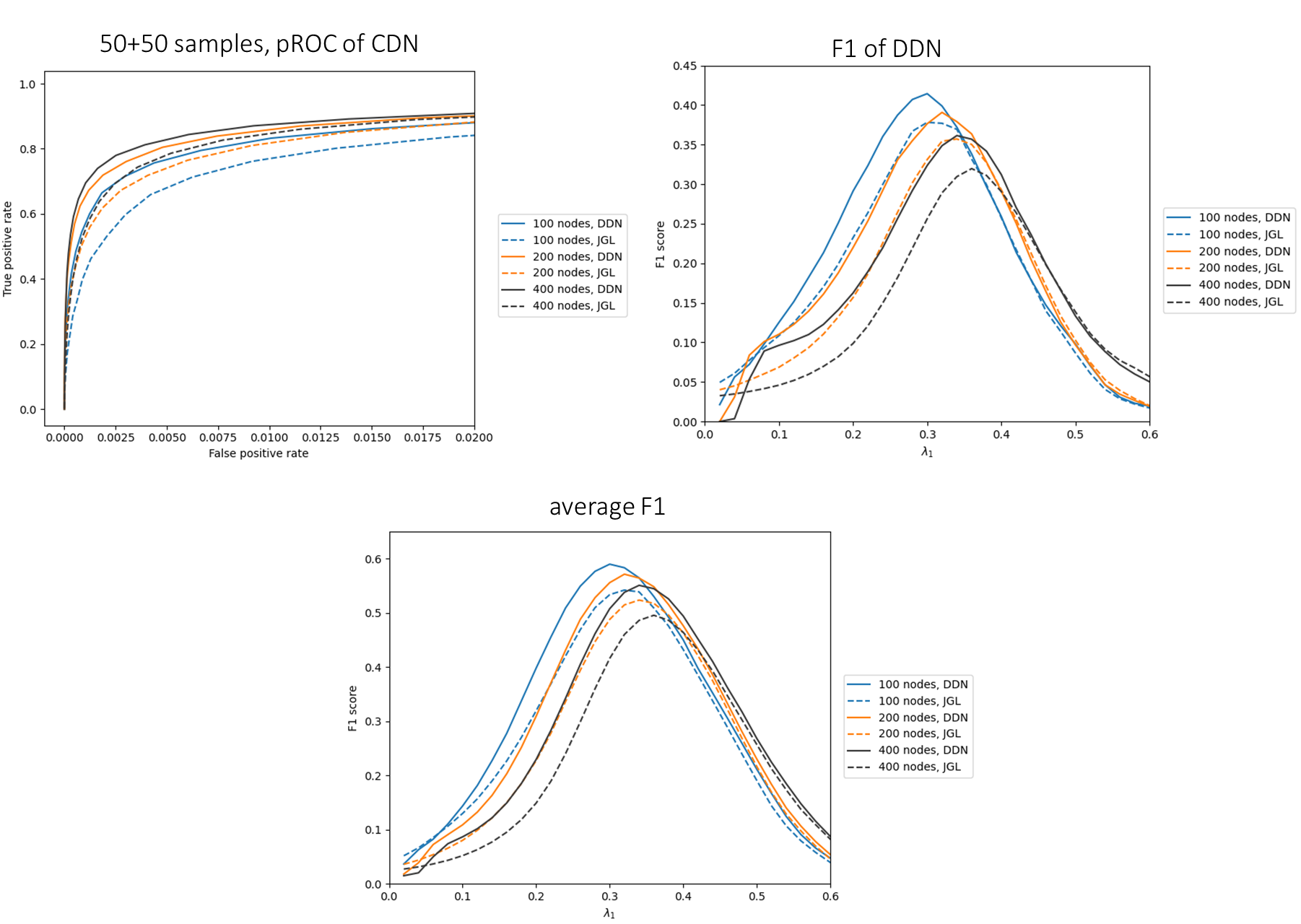


**Figure S9**. Comparative evaluation of DDN3.0 and benchmark JGL measured by pROC curve of the common network, $F_{1}$ of the rewired network, and average $F_{1}$ (sample size 50+50).

## Evaluating performance on the impact of imbalance sample groups.

We also studied the impact of imbalanced sample sizes. We first generated a 100-nodes scale-free graph with two modules and then simulated two scenarios. In the first scenario, we have 50+500 samples, which mimics the case of imbalanced sample sizes. In the second scenario, we generated a balanced 275+275 samples. For both DDN and JGL, we placed the same weight on each group. Both methods perform better with the balanced case, as the differential network becomes especially hard to estimate with smaller samples in one condition. Nonetheless, DDN outperformed JGL (Fig. S10).


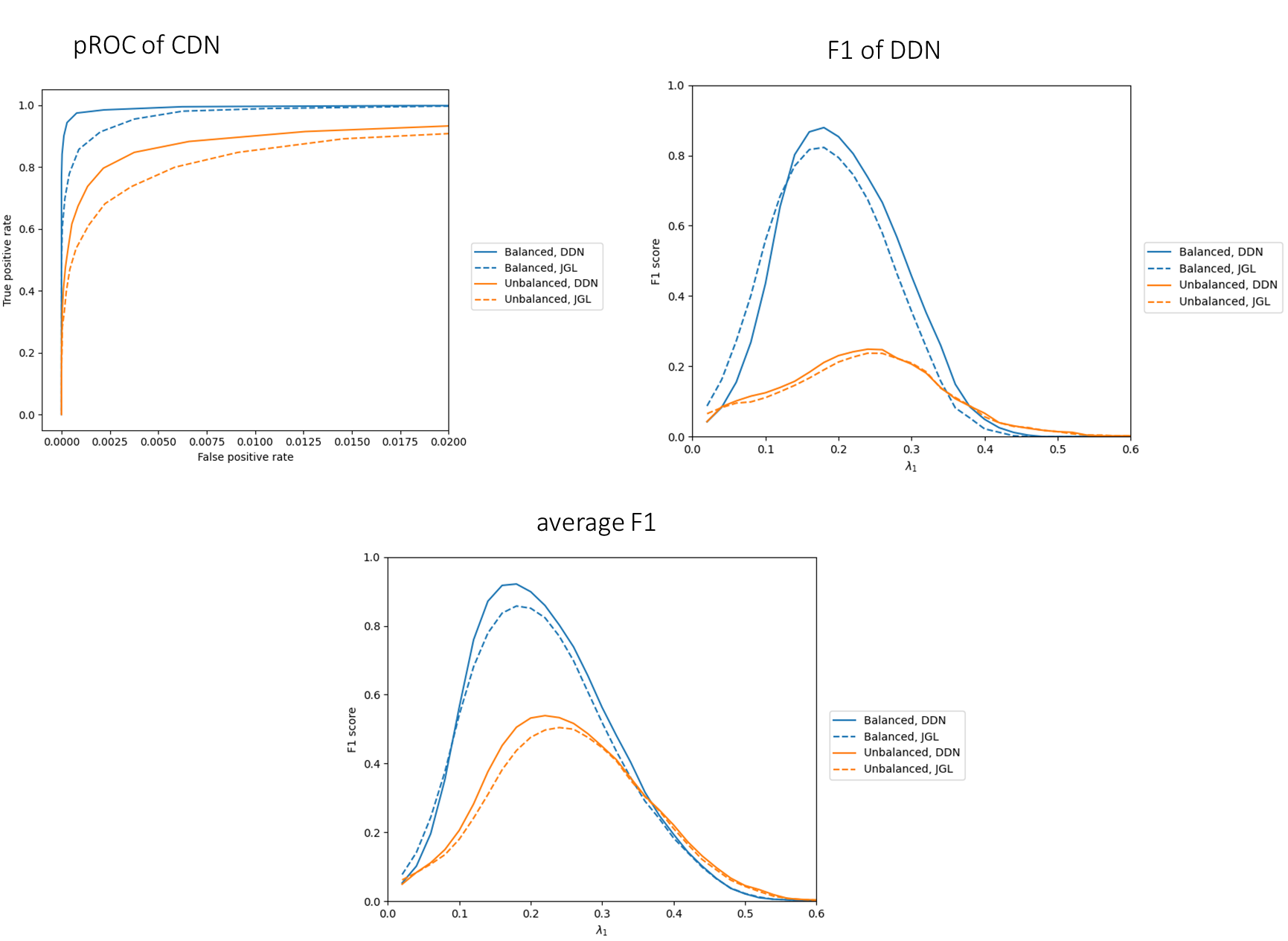


**Figure S10**. Comparative evaluation of DDN3.0 and benchmark JGL measured by pROC curve of the common network, $F_{1}$ of the rewired network, and average $F_{1}$ (balance versus imbalance samples).

## Comparison of DDN3.0, DINGO, and JGL

In addition, we compared DDN3.0 with DINGO and GJL on a random graph. The network is similar to the design used in DINGO’s supplementary section 3.2. We used 200 samples for each condition. $\lambda_{1}$ changed from 0.02 to 1 for DDN3 and JGL, while $\lambda_{2}$ changed from 0 to 0.15 with step 0.025. By default, DINGO estimates its own $\lambda_{1}$ with BIC. We first show results using this setting, where the estimated $\lambda_{1}$ of DINGO is 0.11. We show the precision-recall curves and TP-FP curves for the common network and the differential network (Fig. S11).


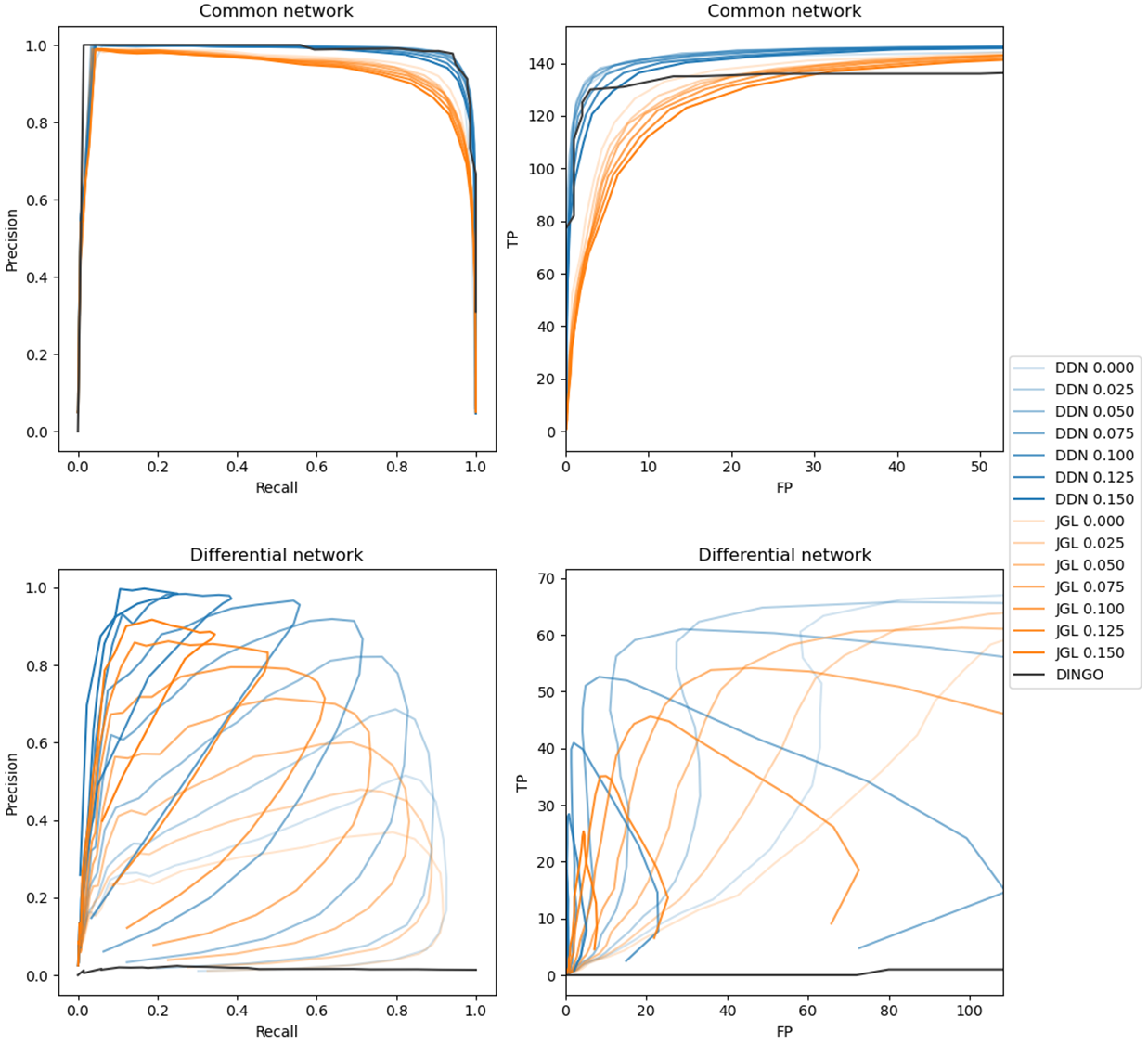


**Figure S11**. Comparative evaluation of DDN3.0 and benchmark DINGO and JGL measured by ROC and PR curves of common and differential networks (random graph, DINGO $\lambda_{1}$ selected by its own).

Note that DINGO and for DDN/JGL generate curves differently. The common network curve for DINGO is obtained by setting different thresholds on its precision matrix, while the differential network curve is from thresholding the DINGO differential score matrix. Each curve for DDN 3.0 and JGL represents a particular value of $\lambda_{2}$, and points in one curve are obtained by scanning different $\lambda_{1}$ values.

DINGO works reasonably well in detecting common networks, but its performance is not good in detecting the structure of differential networks. The main reason is that DINGO attempts to reconstruct the residual covariance matrix by low-rank approximation, which is not suitable for inferring network structures. These observations reflect the different design purposes of DINGO from DDN 3.0 and JGL. Therefore, we did not include DINGO in more comprehensive simulations.


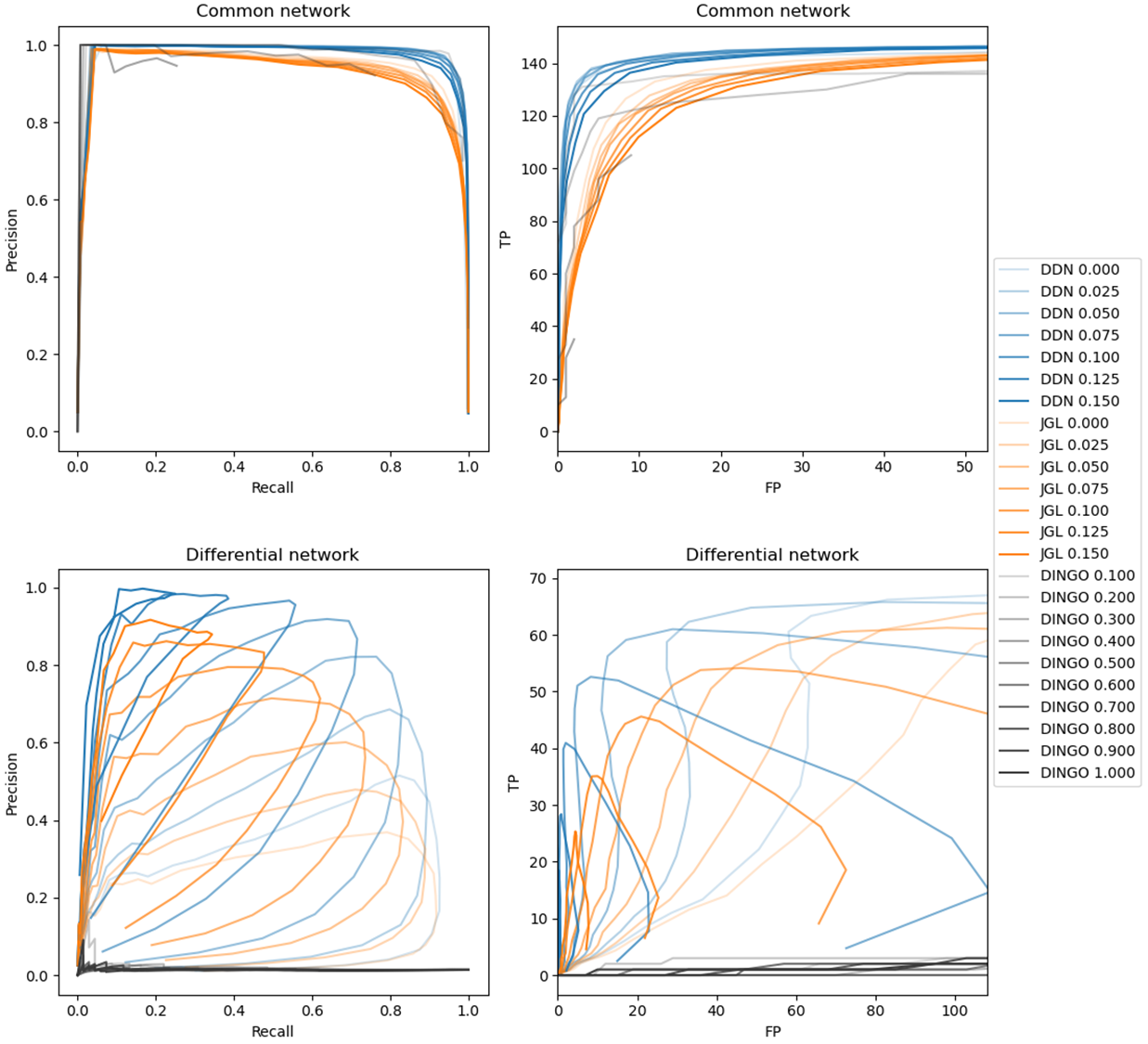


**Figure S12**. Comparative evaluation of DDN3.0 and benchmark DINGO and JGL measured by ROC and PR curves of common and differential networks (random graph).

We also manually surveyed a range of $\lambda_{1}$ from 0.1 to 1.0 with step 0.1 for DINGO. Note that different curves for DINGO are from different $\lambda_{1}$, while the points in each curve are from thresholding the precision matrix or differential score matrix. As before, different curves for DDN 3.0 or JGL represent different $\lambda_{2}$, while the points in each curve are for different $\lambda_{1}$ values. As shown in Fig. S12, the performance of DINGO on detecting differential edges remains unsatisfactory. Also note that with a large $\lambda_{1}$, DINGO does not find any common edge, so there is no dark green curve for the common network figures in Fig. S12.

## Comparison of DDN3.0, DINGO, and JGL on computational efficiency

We first compared DDN 3.0, JGL, and DINGO on a dataset with 100 and 200 nodes (Table S3). It is important to note that the running time for different algorithms at different $\lambda_{1}$ can be quite different due to the iterations needed to converge. In real applications, it is common to scan different $\lambda_{1}$ values. Therefore, in part of our comparisons, we report the timing based on the total time needed for scanning $\lambda_{1}$ values from 0.05 to 1.0, with a step size of 0.05. For all methods involved, warm start was not used. Parallel computing also was not used (except for the DINGO bootstrapping step). All methods were repeated five times for each $\lambda_{1}$. We used the “JGL” function in the JGL R package, the “dingo” function in the iDINGO R package, and the DDN3 Python package. For DDN3.0, we used the residual update acceleration strategy unless otherwise stated. All DDN methods are Numba (Lam, et al., 2015) accelerated (https://numba.pydata.org/). The timing was performed on a desktop computer with Intel Core i-8700 CPU and 32 GB RAM. The operating system was Windows 11. R 4.3.2 and Python 3.11 was used. Each condition had 50 samples for a total of 100 samples. For DINGO, we manually set each $\lambda_{1}$, repeated the bootstrapping 10 times, and ran with 10 cores. As shown in Table S3, the speed of DDN3.0 is much faster than JGL and DINGO, with at least 7 times speed up. We note that the expectation-maximization of DINGO takes a significant amount of time, which each bootstrapping operation needs to repeat, explaining the significantly longer running time of DINGO.

**Table S3**. Computation time comparison between DDN3.0, JGL, and DINGO.

|  | 100 nodes (seconds) | 200 nodes (seconds) |
| --- | --- | --- |
| DDN 3.0 | 0.7 | 2.76 |
| JGL | 4.83 | 17.8 |
| DINGO | 1020.23 | 3200.43 |

We then compared DDN 3.0 with JGL with a scale-free graph with four modules. We used different numbers of nodes and samples for the speed comparison to study the impact of larger networks. We no longer compared DINGO due to its much longer run time. As we can see in Table S4 and Table S5, DDN3.0 is 10 to 20 times faster with larger graph sizes, and 5 to 7 times faster even when the graph is small. Results are reported by summing the running time with different $\lambda_{1}$ values.

**Table S4**. Computation time comparison between DDN3.0 and JGL on larger networks (200+200 samples).

|  | 100 nodes (s) | 200 nodes (s) | 400 nodes (s) |
| --- | --- | --- | --- |
| DDN 3.0 | 0.91 | 2.76 | 10.01 |
| JGL | 4.72 | 17.8 | 111.88 |

**Table S5**. Computation time comparison between DDN3.0 and JGL on larger networks (50+50 samples).

|  | 100 nodes (s) | 200 nodes (s) | 400 nodes (s) |
| --- | --- | --- | --- |
| DDN 3.0 | 0.70 | 2.51 | 10.51 |
| JGL | 4.83 | 23.67 | 189.78 |

The details on the running time of each $\lambda_{1}$ is given in Fig. S13 for graphs with 100, 200, and 400 nodes, and 200 samples. This provides us with a view of the computational time versus both network size and sparsity levels. The running time difference with a smaller $\lambda_{1}$ is clear between DDN3.0 and JGL. The difference is not so clear with larger $\lambda_{1}$, when the estimated network becomes very sparse. In this simulation, a $\lambda_{1}$ of ~0.2 leads to good consistency between ground truth; too large a value misses most edges in the common networks.


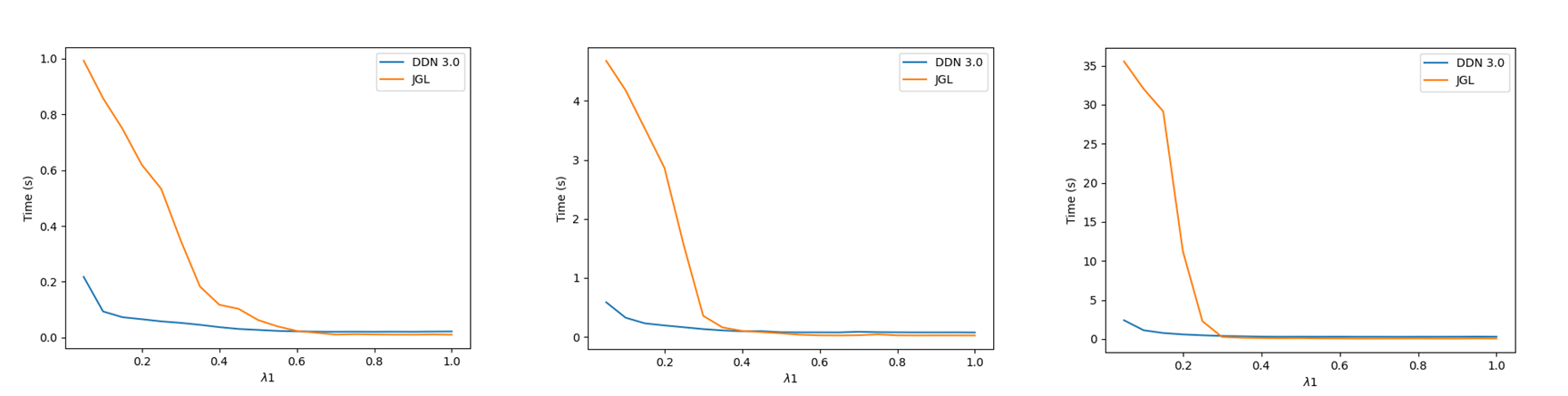


**Figure S13**. Comparative evaluation of the running time of DDN3.0 and JGL with different network sizes and sparsity levels (200 samples; 100/200/400 nodes).

We note that the convergence criteria of DDN and JGL are different, which might influence the running time comparison. DDN uses the absolute change of Lasso coefficient $\beta$, while JGL uses the relative change of the precision matrix. The relative change is not suitable for DDN, as the $\beta$ could easily go zero. The thresholds for both methods are set as ${10}^{-6}$, though they have different meanings.

## Additional comparison of DDN3.0 and DDN2.0 on computational efficiency

DDN 3.0 uses several strategies to improve the speed of DDN 2.0. Among them, the most important are residual update strategy (referred to as “DDN-Resi”) and correlation matrix update strategy (“DDN-Corr”). Here we show some additional comparisons of DDN 2.0 with these two strategies. We first study the impact of feature numbers (Fig. S14). We simulate scale-free graphs with four modules. The node numbers used are 100, 200, 500, 1000, and 2000. Each condition has 100 samples for a total of 200 samples. We fixed $\lambda_{1}$ at 0.3, and $\lambda_{2}$ at 0.1. For a fair comparison, all three methods were Numba accelerated (including DDN 2.0) and repeated 10 times. Then we studied the impact of sample size on computation time; results are shown in Fig. S15. We also compared these three methods under different values of $\lambda_{1}$ (Table S6). Specifically, we show the results with 200 nodes and 100 samples in Fig. S16.


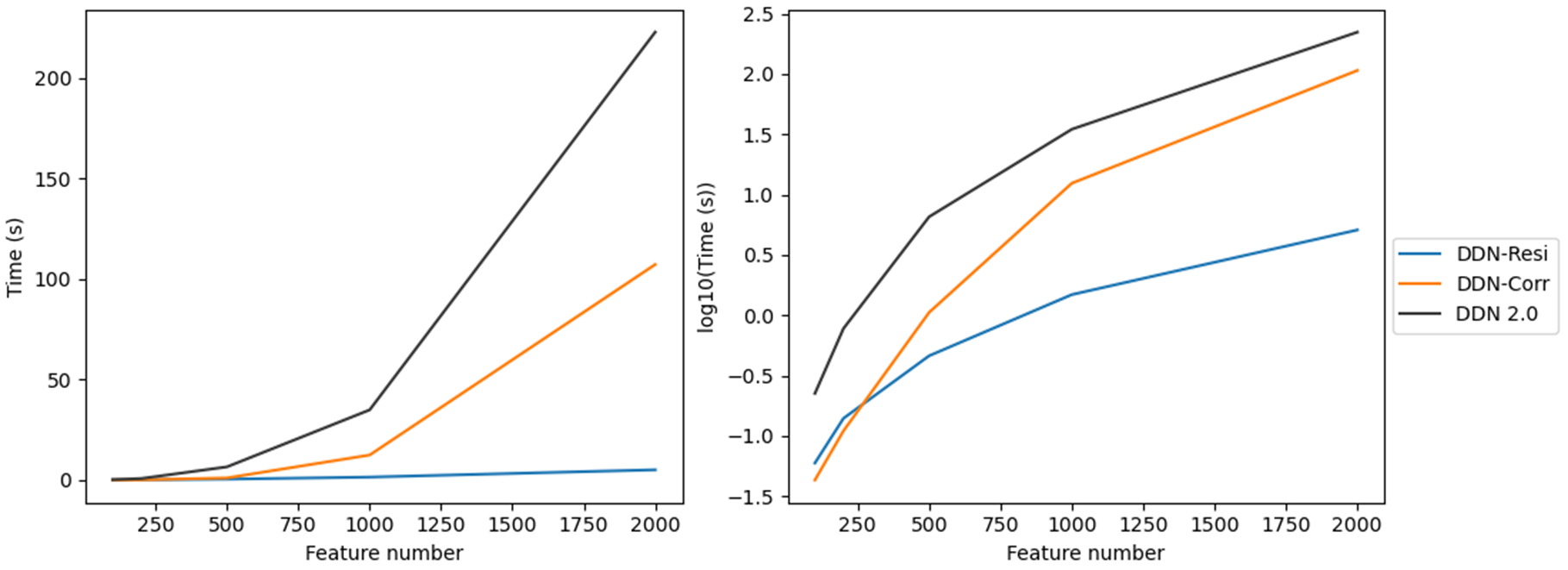


**Figure S14**. Comparative evaluation of DDN3.0 and DDN2.0 (Resi and Corr).


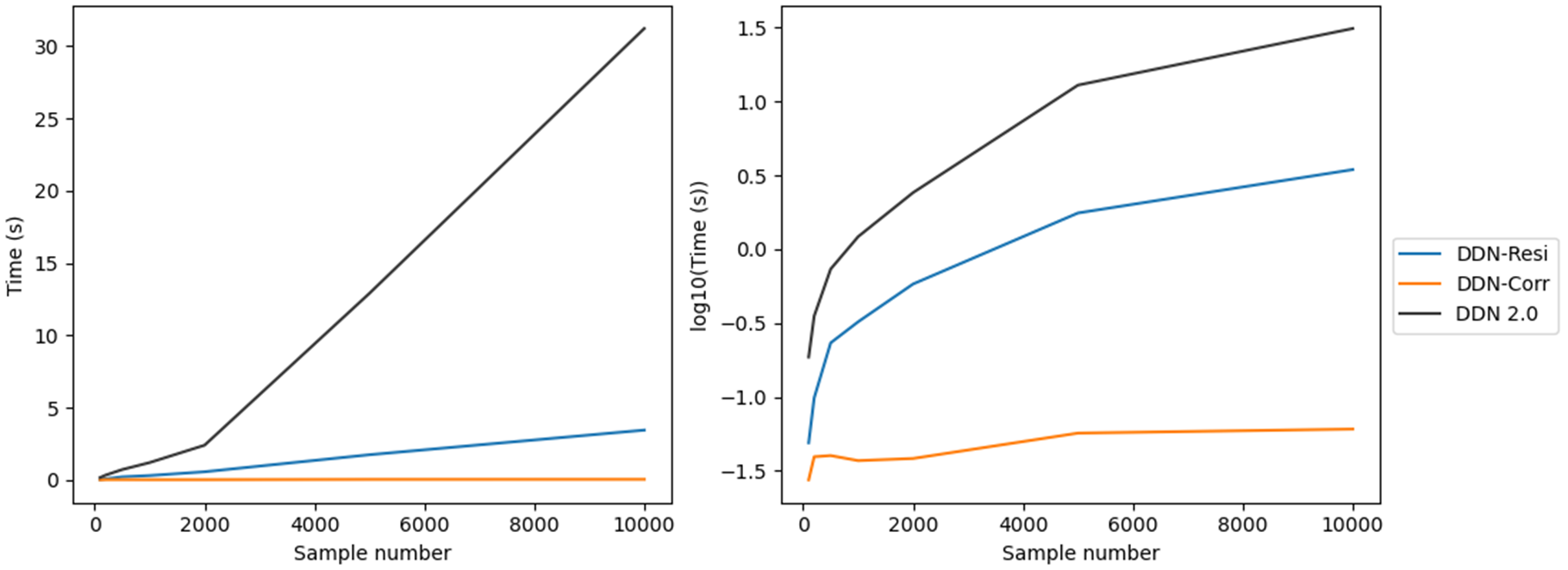


**Figure S15**. Comparative evaluation of DDN3.0 and DDN2.0 (Resi and Corr).

**Table S6**. Comparative evaluation of DDN3.0 and DDN2.0 (under different values of $\lambda_{1}$).

|  | 100 nodes  100 samples (s) | 200 nodes  100 samples (s) | 100 nodes  1000 samples (s) |
| --- | --- | --- | --- |
| DDN-Resi | 0.85 | 2.94 | 2.26 |
| DDN-Corr | 0.85 | 4.40 | 0.52 |
| DDN 2.0 | 3.58 | 24.00 | 9.66 |


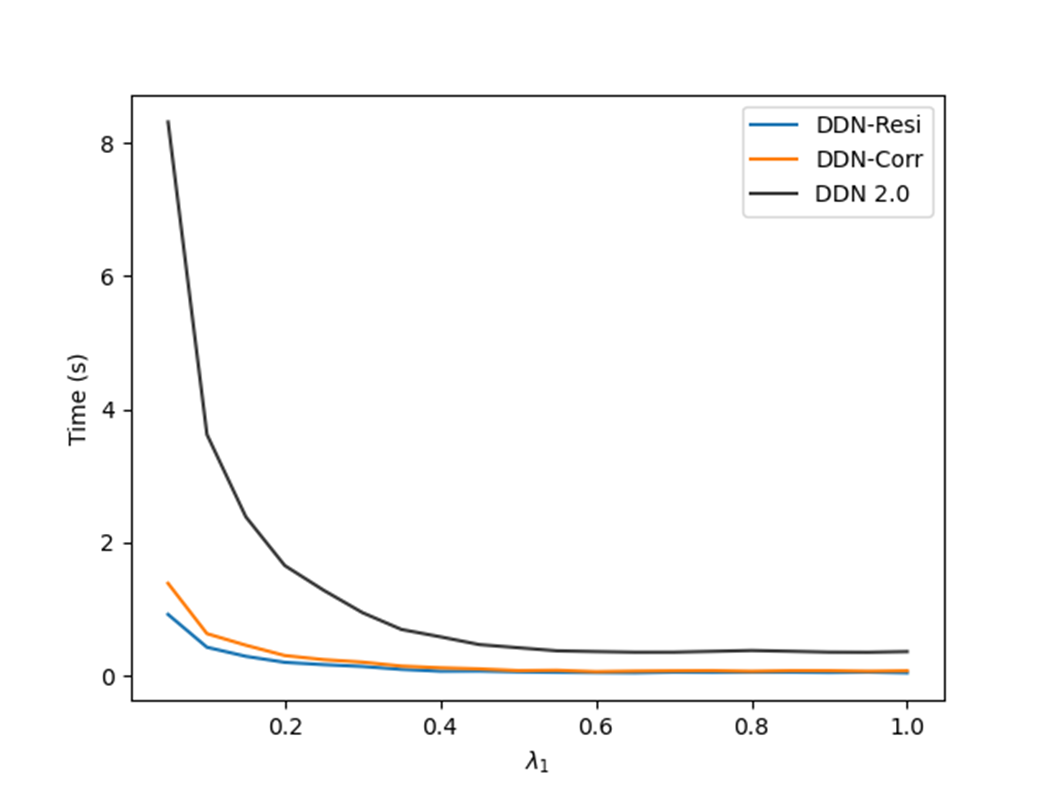


**Figure S16**. Comparative evaluation of DDN3.0 and DDN2.0 (under different values of $\lambda_{1}$).

# Sensitivity of hyperparameter determination

In this section, we assess whether the hyperparameter determination process is sensitive to changes in the dataset and/or modeling assumptions. We considered three types of simulation: edge weight variation in the graph, sample sizes variation of two conditions, and distribution deviation from the normal assumption. The first two types of simulations are related to dataset changes, while the third is related to modeling assumption changes.

We started from an initial scale-free network with 40 edges. To simulate rewiring, we removed ten edges for each condition, and we made sure that an edge removed in condition 1 will not be removed in condition 2. We conducted the four following experiments.

1. Baseline: each edge is assigned a weight of 0.5 or -0.5, each condition contains 100 samples, and all simulated data follows the normal distribution
2. Weight variation of edges: add randomness to edge weights (around baseline). Specifically, a uniform random number ranging from -0.1 to 0.1 is added to the current weight. Otherwise, this experiment is the same as the baseline.
3. Sample size variation: add randomness to the sample sizes for each condition (around baseline). Specifically, the sample size for each condition is uniformly selected between 80 to 120.
4. Distribution that deviates from Gaussian: a mixture of Beta distribution and a smaller amount of Gaussian distribution is used, instead of pure Gaussian. Specifically, we added Beta distributed noise in addition to Gaussian noise in each node of the graph when generating samples. The beta random numbers are generated by the beta function of NumPy. Both the alpha and beta parameters are 0.5. The Gaussian component has a standard deviation of 0.1. After adding these two parts, they are standardized to zero mean and unit variance.

We generated samples using a directed graph based on these settings. As discussed in section 2.4 of the main body, we use two hyper-parameter determining methods: 1) joint grid search CV and 2) using formulas for 𝜆_1_ and 𝜆_2_. For each experiment setting, we repeated 10 times for each hyper-parameter determining method. The experimental results are shown in Fig. S17. For method 1, we observed some small variation in the estimation of 𝜆_1_ and 𝜆_2_. Generally, the estimation of $\lambda_{2}$ showed more variation with respect to the mean value, which reflects the fact that $\lambda_{2}$ estimations are more difficult. Since method 2 is based on two formulas, it will not show any variation unless the sample size changes. These two methods are based on different principles and are expected to give different estimations of the hyper-parameters. In conclusion, these results demonstrate the robustness of the hyperparameter determination methods with respect to changes in the dataset and/or modeling assumptions.


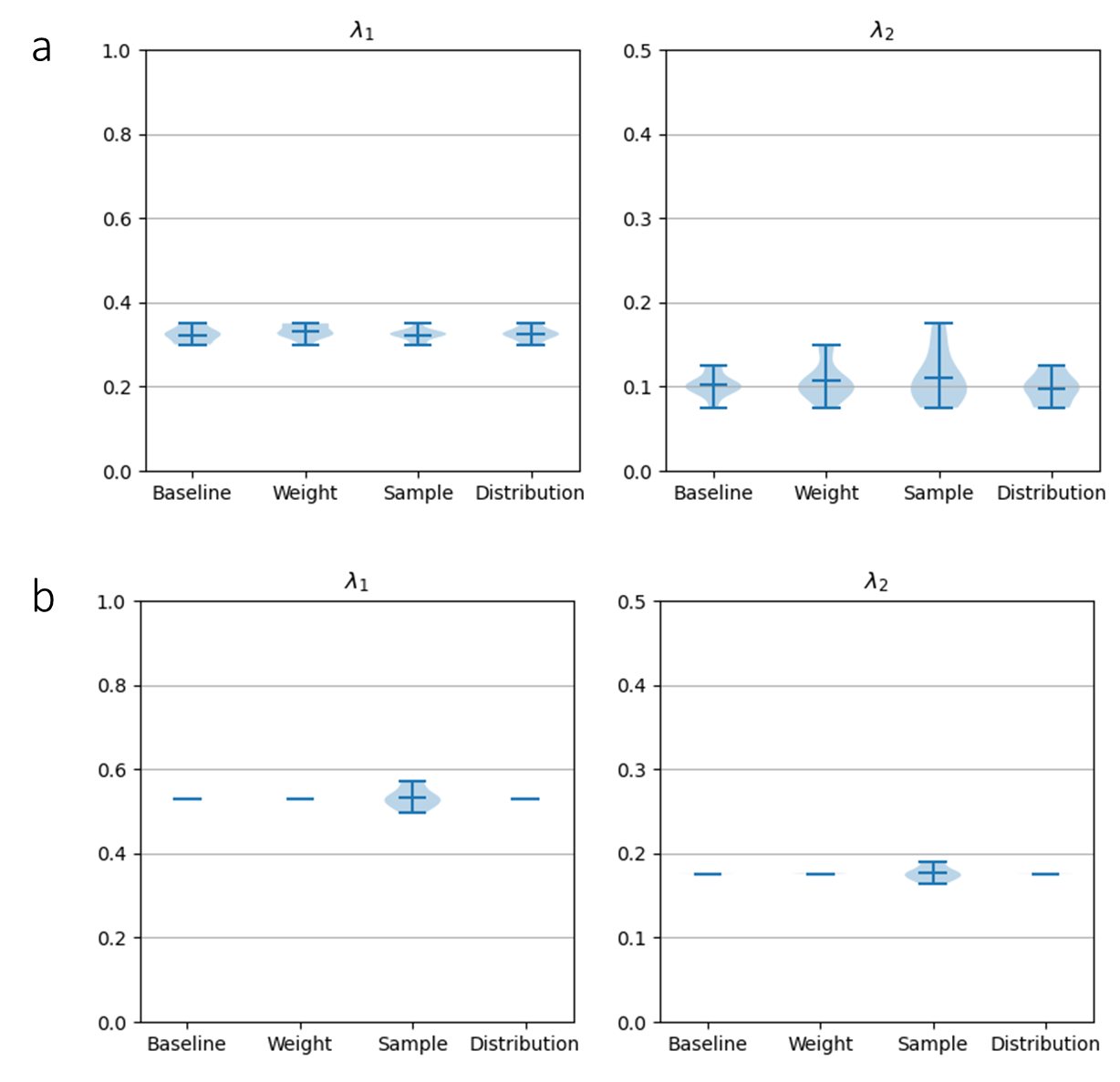


**Figure S17**. Sensitivity assessment of the hyperparameter determination process with respect to changes in the dataset and/or modeling assumptions. (a) Joint estimation (2D grid search with cross-validation). The minimum of the survey surface corresponds to the selected hyperparameter values. (b) Using formulas to directly calculate hyperparameters. In all scenarios, the higher values of hyperparameters lead to sparser common networks, as well as fewer rewired edges. The estimated values are drawn as violin plots. Baseline: baseline experiment. Weight: weight variation of edges. Sample: sample size variation. Distribution: distribution that deviates from Gaussian.

# Prototype of the application on multi-omics data

We prototyped an integrative-DDN (iDDN) tool to integrate multi-omics data for modeling biological systems. Such an extension introduces new opportunities including detecting both intra- and inter-omics network structure and its rewiring, which can provide a more comprehensive understanding of the regulatory networks involved in disease progression.

Here we present a preliminary application of iDDN that integrates transcription factor (TF) proteomics data and mRNA data to learn common and differential networks of two forms of ovarian cancer with very different prognoses. Data sets were obtained from the CPTAC-OV2 project (Zhang H, et al., 2016), which aims to perform a comprehensive proteomic and genomic characterization of human ovarian high-grade serous carcinomas. For proteomics expression, we selected 83 samples that were obtained from ovarian cancer tumors. The protein expression matrix was normalized by total expression quantity. Proteins with >10% missing rates in the 83 samples were removed and the remaining missing values were imputed with the mean expressions. By searching the NCI database, we found 82 of the 83 samples have matching RNA-seq profiles. Tumor samples with somatic mutations on the genes of BRCA1/BRCA2/PTEN are identified as homologous recombination deficiency (HRD). 19 tumor samples were marked as HRD+ and 63 samples as HRD-. We obtained a list of 171 HRD-associated genes from CPTAC-OV1; 120 genes overlap both with the gene list of copy number data and the gene list of mRNA expression data. These genes were further filtered by the protein list and searched through the TRRUST online database (Han, 2017). We selected 23 up-regulating TFs, where each regulates at least three of the 120 genes with a p-value<0.005.

Representative preliminary results of iDDN are reported in Fig. S18. Under a typical set of hyperparameters ($\lambda_{1}=0.25$, $\lambda_{2}=0.05$), iDDN detected 289 common edges among mRNAs and TFs, and 496 differential edges between HRD+ and HRD- conditions. Further studies are needed to refine and interpret these preliminary results, which may provide insights into the mechanisms behind the impact of homologous recombination deficiency for the progression of ovarian cancer and the survival of patients.


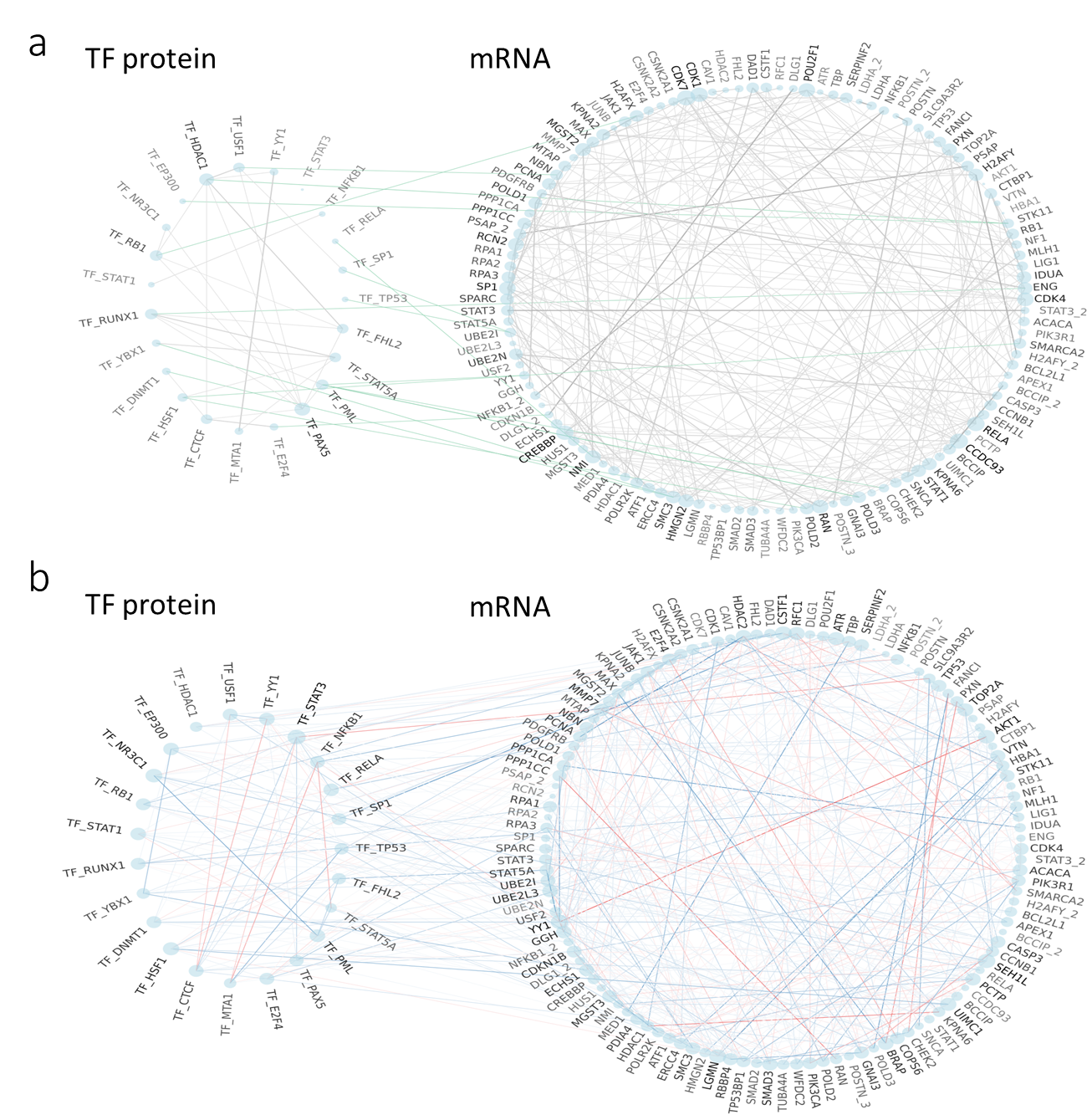


**Figure S18**. Preliminary results of iDDN on the CPTAC-OV2 prospective data. We use 23 TF proteins and 120 mRNAs in this analysis. There are 19 samples for condition 1 (HRD+) and 63 for condition 2 (HRD-), with parameter setting 𝜆_1_=0.25 and 𝜆_2_=0.05. (a) iDDN detects 289 common edges. The edges among TFs and those among mRNAs are shown in black, while the edges across omics types are shown in green. The absolute value of the weight of each edge is represented by the thickness of edge. The size of each node represents the degree of that node. (b) iDDN detects 496 differential edges. The 339 differential edges for HRD+ are shown in blue, and the 157 for HRD- are shown in red.

# Discussion

In this Application Note, we proposed, evaluated, and applied several methodological and algorithmic improvements to the DDN framework. The resulting DDN3.0 performs differential network analysis, with a more efficient and accurate inference of common and rewired network structures.

In addition to the limitations and future work discussed in the “Discussion” section of the main text, we note two additional limitations on the current DDN3.0 framework that require further improvement. First, genes often work together to achieve a complex biological function but collinearity among the predictors remains a theoretically unresolved problem. One practical solution is to perform gene clustering and select the ‘tag genes’ as the input to DDN3.0. Once the dependency networks are constructed, the hub nodes then return all associated genes for the enrichment analysis. Alternatively, recall that the fused Lasso-regression performs simultaneous variable selection and coefficient estimation to infer conditional dependency. Thus, once hubs of dependency networks are identified, we can recruit the 'highly correlated' genes (genes in collinearity and thus numerically excluded from the sparse solution) for enrichment analysis. Second, DDN3.0 currently takes bulk data to infer common and rewired regulatory network structures but network rewiring may only occur in some cellular subpopulations in the bulk sample. In such a case, we could perform sample-wise deconvolution or residualization prior to the DDN analysis.

# Python Package

The Python package of DDN3.0 is freely available at <https://github.com/cbil-vt/DDN3> under the MIT license**.** The package is stored and archived at <https://pypi.org/project/ddn3/>. Tutorials and detailed documentations are provided at <https://ddn-30.readthedocs.io/en/latest/index.html>.

# References

Bandyopadhyay, S.*, et al.* Rewiring of genetic networks in response to DNA damage. *Science* 2010;330(6009):1385-1389.

Banerjee, O., Ghaoui, L.E. and d’Aspremont, A.J.J.o.M.l.r. Model selection through sparse maximum likelihood estimation for multivariate Gaussian or binary data. 2008;9(Mar):485-516.

Barabasi, A.L., Gulbahce, N. and Loscalzo, J. Network medicine: a network-based approach to human disease. *Nature reviews. Genetics* 2011;12(1):56-68.

Califano, A. Rewiring makes the difference. *Molecular systems biology* 2011;7:463.

Chen, C.*, et al.* Two gene co-expression modules differentiate psychotics and controls. *Mol Psychiatry* 2013;18(12):1308-1314.

Chen, L.*, et al.* Unsupervised Deconvolution of Dynamic Imaging Reveals Intratumor Vascular Heterogeneity and Repopulation Dynamics. *PloS one* 2014;9(11):e112143.

Class, C.A.*, et al.* iDINGO-integrative differential network analysis in genomics with Shiny application. *Bioinformatics* 2018;34(7):1243-1245.

Colantuoni, C.*, et al.* Temporal dynamics and genetic control of transcription in the human prefrontal cortex. *Nature* 2011;478(7370):519-523.

Creixell, P.*, et al.* Navigating cancer network attractors for tumor-specific therapy. *Nat Biotechnol* 2012;30(9):842-848.

Danaher, P., Wang, P. and Witten, D.M. The joint graphical lasso for inverse covariance estimation across multiple classes. *J R Stat Soc Series B Stat Methodol* 2014;76(2):373-397.

Friedman, J., Hastie, T. and Tibshirani, R. The elements of statistical learning. Springer series in statistics New York; 2017.

Friedman, J., Hastie, T. and Tibshirani, R.J.B. Sparse inverse covariance estimation with the graphical lasso. 2008;9(3):432-441.

Ha, M.J., Baladandayuthapani, V. and Do, K.A. DINGO: differential network analysis in genomics. *Bioinformatics* 2015;31(21):3413-3420.

Han, H., et al. TRRUST v2: an expanded reference database of human and mouse transcriptional regulatory interactions. 2017;46(D1):D380-D386

He, H.*, et al.* A Statistical Test for Differential Network Analysis Based on Inference of Gaussian Graphical Model. *Scientific reports* 2019;9(1):10863.

Herrington, D.M.*, et al.* Proteomic Architecture of Human Coronary and Aortic Atherosclerosis. *Circulation* 2018;137(25):2741-2756.

Herrington, D.M.*, et al.* Proteomic architecture of human coronary and aortic atherosclerosis. 2018;137(25):2741-2756.

Hu, J.X., Thomas, C.E. and Brunak, S. Network biology concepts in complex disease comorbidities. *Nature reviews. Genetics* 2016;17(10):615-629.

Kang, H.J.*, et al.* Spatio-temporal transcriptome of the human brain. *Nature* 2011;478(7370):483-489.

Kuhn, A.*, et al.* Population-specific expression analysis (PSEA) reveals molecular changes in diseased brain. *Nature methods* 2011;8(11):945-947.

Lam, S.K., Pitrou, A. and Seibert, S. Numba: A llvm-based python jit compiler. In, *Proceedings of the Second Workshop on the LLVM Compiler Infrastructure in HPC*. 2015. p. 1-6.

Mitra, K.*, et al.* Integrative approaches for finding modular structure in biological networks. *Nature reviews. Genetics* 2013;14(10):719-732.

Ottenbros, I.*, et al.* Network Analysis to Identify Communities Among Multiple Exposure Biomarkers Measured at Birth in Three Flemish General Population Samples. *Front Public Health* 2021;9:590038.

Reverter, A.*, et al.* Regulatory impact factors: unraveling the transcriptional regulation of complex traits from expression data. *Bioinformatics* 2010;26(7):896-904.

Tian, Y.*, et al.* KDDN: an open-source Cytoscape app for constructing differential dependency networks with significant rewiring. *Bioinformatics* 2015;31(2):287-289.

Tian, Y.*, et al.* Knowledge-fused differential dependency network models for detecting significant rewiring in biological networks. *BMC systems biology* 2014;8(1):87.

Tian, Y.*, et al.* Knowledge-guided differential dependency network learning for detecting structural changes in biological networks. In, *ACM International Conference on Bioinformatics and Computational Biology*. 2011. p. 254-263.

Tibshirani, R.*, et al.* Strong rules for discarding predictors in lasso-type problems. *J R Stat Soc Series B Stat Methodol* 2012;74(2):245-266.

Tseng. Convergence of a block coordinate descent method for nondifferentiable minimization. *Journal of optimization theory applications* 2001;109(3):475-494.

Wang, Y.X.R.*, et al.* Network Modeling in Biology: Statistical Methods for Gene and Brain Networks. *Stat Sci* 2021;36(1):89-108.

Zhang, B.*, et al.* Differential Dependency Network Analysis to Identify Condition-Specific Topological Changes in Biological Networks. *Bioinformatics* 2009;25(4):526-532.

Zhang, B.*, et al.* DDN: a caBIG(R) analytical tool for differential network analysis. *Bioinformatics* 2011;27(7):1036-1038.

Zhang, B., Tian, Y. and Zhang, Z. Network biology in medicine and beyond. *Circulation. Cardiovascular genetics* 2014;7(4):536-547.

Zhang, B. and Wang, Y. Learning structural changes of Gaussian graphical models in controlled experiments. In, *Uncertainty in Artificial Intelligence (UAI 2010)*. 2010.

Zhang, H.*, et al.* Integrated proteogenomic characterization of human high-grade serous ovarian cancer. *Cell* 2016;166(3):755-765.

Zhao, S.D., Cai, T.T. and Li, H. Direct estimation of differential networks. *Biometrika* 2014;101(2):253-268.

Zhao, T.*, et al.* The huge Package for High-dimensional Undirected Graph Estimation in R. *J Mach Learn Res* 2012;13:1059-1062.
